# Socioeconomic inequalities in co-morbidity of overweight, obesity and mental ill-health from adolescence to mid-adulthood in two national birth cohort studies

**DOI:** 10.1016/j.lanepe.2021.100106

**Published:** 2021-04-30

**Authors:** Amal R. Khanolkar, Praveetha Patalay

**Affiliations:** a MRC Unit for Lifelong Health and Ageing at UCL, 1-19 Torrington Place, WC1E 7HB London, United Kingdom; b Institute for Environmental Medicine, Karolinska Institutet, Stockholm, Sweden; c Centre for Longitudinal Studies, UCL, London, United Kingdom

**Keywords:** Socioeconomic inequalities, Socioeconomic position, Comorbidity, Obesity, Mental health, Depression

## Abstract

**Aim:**

To examine socioeconomic inequalities in comorbidity risk for overweight (including obesity) and mental ill-health in two national cohorts. We investigated independent effects of childhood and adulthood socioeconomic disadvantage on comorbidity from childhood to mid-adulthood, and differences by sex and cohort.

**Methods:**

Data were from 1958 National Child Development Study (NCDS58) and 1970 British Cohort Study (BCS70) [total N=30,868, 51% males] assessed at ages 10, 16, 23/26, 34 and 42 years. Socioeconomic indicators included childhood and adulthood social class and educational level. Risk for i. having healthy BMI and mental ill-health, ii. overweight and good mental health, and iii. overweight and mental ill-health was analysed using multinomial logistic regression.

**Findings:**

Socioeconomic disadvantage was consistently associated with greater risk for overweight-mental ill-health comorbidity at all ages (RRR 1.43, 2.04, 2.38, 1.64 and 1.71 at ages 10, 16, 23, 34 and 42 respectively for unskilled/skilled vs. professional/managerial class). The observed inequalities in co-morbidity were greater than those observed for either condition alone (overweight; RRR 1.39 and 1.25, mental ill-health; 1.36 and 1.22 at ages 16 and 42 respectively, for unskilled/skilled vs. professional/managerial class). In adulthood, both childhood and adulthood socioeconomic disadvantage were independently associated with comorbid overweight-mental ill-health, with a clear inverse gradient between educational level and risk for comorbidity. For instance, for the no education group (compared to university education) the RRR is 6.11 (95% CI 4.31-8.65) at age 34 and 4.42 (3.28-5.96) at age 42. There were no differences observed in the extent of inequalities by sex and differences between cohorts were limited.

**Interpretation:**

While socioeconomic disadvantage in childhood and adulthood are consistently and independently associated with greater risk for mental ill-health and being overweight separately, these associations are even larger for their comorbidity across the lifecourse. These findings are significant given the increasing global prevalence of obesity and mental ill-health, and their implications for lifelong health and mortality.

**Funding:**

This research was supported by grants from the Wellcome Trust (ISSF3/ H17RCO/NG1) and Medical Research Council (MRC) [MC_UU_00019/3].

**Research in context**

**Evidence before this study**

Socioeconomic inequalities in overweight/obesity and mental ill-health are well described. There is also substantial evidence for comorbidity (i.e. co-occurrence) of obesity and mental ill-health in adolescence and across adulthood. However, it is known whether the prevalence of comorbidity differs between socioeconomic groups and across the lifecourse.

**Added value of this study**

This study found that socioeconomic disadvantage was consistently associated with increased risk of comorbidity between obesity and mental ill-health in childhood and adolescence, and the risk progressively increased with age. Greater levels of socioeconomic disadvantage were associated with increasing risks of comorbidity, with inverse gradients observed with childhood and adulthood social class and educational level. Additionally, socioeconomic disadvantage in childhood was independently associated with comorbidity in adulthood.

**Implications of all the available evidence**

This study highlights socioeconomic inequalities in two health conditions that contribute significantly to global disease burden. While inequalities in either condition alone are well established, this study highlights the need for comprehensive public health interventions and policies designed across the lifecourse including childhood if we are to effectively address this comorbidity.

Alt-text: Unlabelled box

## 1 Introduction

Obesity and mental ill-health are often childhood-onset, are chronic with a strong propensity to track across the lifecourse, and are associated with increased risk for a wide range of health outcomes and mortality [1,2]. Both conditions have increased in prevalence globally and contribute significantly to global disease burden [[3], [4], [5]] Additionally, separately both obesity and mental ill-health are more common among socioeconomically disadvantaged individuals [[6], [7], [8], [9]]. There is substantial evidence for comorbidity between being obese and greater common mental health difficulties (like depression and anxiety) [[10], [11], [12], [13], [14]].

Given the strong socioeconomic patterning in obesity and mental ill-health across the lifecourse, it is possible that socioeconomically disadvantaged individuals are at higher risk of comorbidity between the two conditions (above and beyond the risk of either on its own). In line with increasing prevalence and inequalities with age, it is also possible that the inequalities in the comorbidity also widens with age. Comorbidity is potentially more common in younger generations who experience higher rates of obesity and mental ill-health earlier in life [15,16]. This is substantiated by evidence showing inequalities in childhood obesity have widened across generations in the last few decades, which might result in inequalities in co-morbidities not being the same across generations [17,18]. There is also some limited evidence that women are at higher risk for comorbid obesity and mental ill-health, but there is little known on whether disadvantaged women are more likely to experience higher rates of comorbidity [19,20]. Disadvantaged socioeconomic circumstances in childhood can have long-lasting impacts on health across the life course including obesity and mental ill-health [21,22]. Analysing the independent effects of childhood and adulthood socioeconomic circumstances on obesity and mental ill-health comorbidity helps to understand the longer term impacts of childhood disadvantage and could guide shaping future public health interventions. If childhood socioeconomic disadvantage is found to be independently associated with greater comorbidity risk in adulthood it would further highlight the need for comprehensive policies (health, economic and social) to reduce the negative lifelong impacts of childhood socioeconomic deprivation.

The limited evidence on higher rates of obesity and mental ill-health comorbidity in socioeconomically disadvantaged groups are from cross-sectional studies in adulthood [2,14]. No study has comprehensively examined socioeconomic inequalities between the two conditions using multiple socioeconomic indicators, and if it changes across the lifecourse. If socioeconomic inequalities in comorbidity between obesity and mental ill-health exist, then the health, economic consequences, and additional burden on the public healthcare systems would be much larger than that posed by either condition alone. Importantly, comorbidity between the two conditions is likely to increase with age, as obesity increases across adulthood. In addition, increases in either condition can lead to a rise in the other over time (negative feedback loop) [13], which highlights the importance of studying comorbidity in the same individuals across the lifecourse. This could also provide further explanation for existing and persistent inequalities in morbidity and mortality observed globally.

There have been substantial increases in obesity and, and to a lesser degree in mental ill-health in younger generations in recent decades, including some evidence for earlier onset of both conditions in childhood [17,18,23,24]. Additionally, the increases in prevalence of obesity and mental ill-health have been disproportionately higher in socioeconomically disadvantaged groups, highlighting increasing inequalities [17]. Hence, including two cohorts representing different generations in this study enables us to examine whether socioeconomic inequalities in risk of comorbidity has increased with time (i.e. varies between cohorts). It also allows us to examine whether inequalities start earlier and/or are more pronounced in childhood in recent cohorts.

This study investigated childhood and adulthood socioeconomic inequalities in comorbidity of obesity and mental ill-health using longitudinal data from two large nationally representative birth cohorts initiated 12 years apart. This enabled examining change in socioeconomic inequalities in comorbidity from childhood to mid-adulthood. Additionally, this study also examined sex and cohort differences in the socioeconomic patterning of comorbidity at different ages.

## 2 Methods

### 2.1 Data, setting and study population

For this study, we utilised data from two ongoing national birth cohort studies: the 1958 National Child Development Study (NCDS58) and the 1970 British Cohort Study (BCS70) [25,26]. Both studies were initiated as birth surveys and are prospective and longitudinal; the NCDS58 follows the lives of an initial 17 415 people born in a single week in March 1958 and the BCS70 follows 17 198 people born in a single week in April 1970 in England, Scotland and Wales. More information on the two cohorts can be found at: https://cls.ucl.ac.uk/cls-studies/. Data for this study was drawn from 5 waves at which BMI and mental health symptoms were both assessed; ages 11, 16, 23, 33, and 42 in NCDS58 and ages 10, 16, 26, 34, and 42 in BCS70 (here after ages 10, 16, 23, 34, and 42 for simplicity). Eligible participants were those with a minimum of one datapoint on BMI or mental health recorded at any one of the 5 waves, yielding a final study sample of N=30 868 (NCDS58, N=16 464 and BCS70, N=14 404, 95% and 84% of the initial cohorts respectively, Supplemental Figure 1).

#### 2.1.1 Mental health

Mental ill-health is based on measures of psychological distress which assesses symptoms of common mental health disorders such as anxiety and depression. In this study, we used the parent-reported Rutter internalising scale in childhood and the Malaise inventory in adulthood. The Rutter internalising scale consists of five items describing depressive and anxiety symptoms measured consistently in childhood (ages 10 and 16) and in both cohorts which can be combined to give an index of emotional difficulties in children (for example ‘*Often worried, worries about many things’*) [27]. Parents were asked to indicate whether each description ‘does not apply’, ‘applies somewhat’ or ‘definitely applies’ to a child. The Rutter scale has been extensively validated as a screening and research instrument to detect emotional/behavioural difficulties in children [28].

The 9-item Malaise Inventory (a set of ‘yes-no’ self-completion questions like ‘*Do you often feel miserable or depressed?’*) measures levels of psychological distress including symptoms of anxiety and depression in adults and was measured consistently in all adulthood sweeps in both cohorts (ages 23, 34 and 42) is used here [29]. The measure has been shown to have good psychometric properties in these two cohorts and measurement invariance across ages [30]. Items from the Rutter scale and Malaise inventory are listed in Supplementary Table 1.

Overall psychological distress (symptoms of depression and anxiety) scores were calculated at each age by aggregating the item responses to either the Rutter scale (in childhood) or Malaise Inventory (in adulthood) and creating a total score used in analysis (with higher scores indicating higher levels of psychological distress). These overall scores were dichotomized based on an established cut-off score≥4 for the Malaise inventory to identify individuals with and without high levels of depression and anxiety [31]. There is no established cut-off point for dichotomising the Rutter scale. Based on previous research we chose the 85^th^ percentile as the cut-off point (which corresponds to scores ≥5 and ≥4 for ages 10 and 16 respectively). In both dichotomised variables, those below the threshold cut off, were coded as zero (i.e. having no or low psychological distress and hence ‘good mental health’). Whereas, individuals with total scores above the threshold value were coded as ‘1’ (i.e. having high symptoms of psychological distress or mental ill-health).

#### 2.1.2 Body mass index

BMI [weight (kg)/height (m)^2^] was calculated from objectively assessed height and weight (or self-reported when objective measures were missing at ages 26, 34 and 42 in BCS70 and ages 23 and 42 in NCDS58). Overweight and obesity in childhood were defined using the age- and sex-specific cut-offs proposed by International Obesity Task Force (IOTF) [32]. The WHO criteria of 25-29.99kg/m^2^ and ≥30kg/m^2^ were used for calculating overweight and obesity respectively in adulthood. In all analyses the overweight and obese groups were combined (referred as overweight hereafter) and compared to the healthy weight group. Due to the very small proportions of participants in the underweight group ([<18.5kg/m2], for example <5% in both cohorts in all adulthood sweeps), this category was combined with the normal weight group.

Comorbidity indicator (study outcome): The outcome of interest was created separately at each age by combining the dichotomised BMI and mental health variables resulting in a variable with four categories: 1. healthy BMI and good mental health (reference group [↑BMI-MH↑], 2. healthy BMI and mental ill-health [↑BMI-MH↓], 3. overweight or obesity and good mental health [↓BMI-MH↑], and 4. overweight or obesity and mental ill-health [↓BMI-MH↓].

#### 2.1.3 Socioeconomic indicators

Our predictors of interest included social class (father's social class measured at age 10 and participant's own social class measured at age 42), and highest educational level (ascertained at age 33). To ensure cross-cohort comparability, the UK Registrar General's Social Class (RGSC) 1990 version was used to classify both parental and participant's own social class. The original 6 categories were grouped as follows for analysis: 1. Professional & managerial (reference category and most advantaged group), 2. Non-manual, 3. Manual and 4. Partly skilled & unskilled. The RGSC is one of the most widely used measures of social class in the UK, and has been extensively used in research on health inequalities [33]. The RGSC is largely based on occupations ranked according to skill [33]. Educational levels are based on the highest UK National Vocational Qualifications (NVQ) level (listed in Supplemental Table 3) and included six categories: no education, NVQ level 1 which represents General Certificate of Secondary Education grade D-G or lower; NVQ level 2, General Certificate of Secondary Education grades A*-C and equivalent qualifications; NVQ level 3, A-levels and equivalent; NVQ level 4, earning a degree; and NVQ level 5, university degree or diploma (reference category and most advantaged group). Childhood and adulthood social class and education were harmonised across both cohorts ensuring comparability [34].

## 3 Data analysis

Initial analysis included descriptive statistics of BMI and mental ill-health at each age including means, variances and distributions for continuous measures and prevalence of overweight/obesity and mental ill-health (high psychological distress) in the entire study sample and by cohort and sex. We also estimated mean differences in BMI and mental health by socioeconomic indictors in the pooled study sample (i.e. both NCDS58 and BCS70 cohorts) and by cohort. This was to separately examine the inequality gradients in BMI and mental ill-health at each of the five ages, and how the absolute difference between the lowest and highest socioeconomic categories changed with age, and between the two cohorts.

We used multivariable multinomial logistic regression to estimate relative risk ratios (RRR) of i. ↑BMI-MH↓ ii. ↓BMI-MH↑ and iii. ↓BMI-MH↓ with healthy BMI and good mental health as the baseline, comparing more socioeconomically disadvantaged participants with the most advantaged. The multinomial logistic regression model calculates the relative risk ratio (RRR), which is the ratio of two relative risks (derived from the exponentiated multinomial logit coefficient) and is interpreted for a unit change in the predictor variables [35].

The following models were run separately at the different age sweeps in the pooled cohort to assess RRR for the different categories of the outcome variable across the categories of socioeconomic predictors: ages 10, 16 and 23; associations with childhood social class, age 34; associations with childhood social class and educational level, age 42; associations with childhood social class, educational level and adulthood social class. All models were adjusted for sex and cohort (to adjust for potential differences in the association between socioeconomic predictors and obesity-mental health comorbidity between the two cohorts). This set of main regression modelling was also run in complete cases for comparison.

We also examined associations between childhood social class and risk for the different categories of the comorbidity indicator at each age (to help understand the independent effect of early life social class on comorbidity in both childhood and adulthood). These models were only adjusted for sex.

We tested whether the above associations between the three socioeconomic indicators and the outcome variable differed by 1. cohort and 2. sex by including interactions terms between each socioeconomic indicator and cohort or sex. These interactions tests were run to examine whether either sex or one of the cohorts were at increased risk for socioeconomic inequalities in comorbidity. Interactions tests between sex and socioeconomic indictors were not significant and results are not presented.

Missing data in the five sweeps in both cohorts was addressed using multiple imputation with chained equations assuming data missing at random (MAR – and including 25 imputations). The MAR mechanism (often largely untestable) implies that systematic differences between the missing values and the observed values can be explained by observed data [36], which is a plausible assumption in the British birth cohorts given the rich data available from birth. [37] The main purpose of imputation was to address missing data in BMI and mental health indicators (Rutter and Malaise scores). As long as a study participant had at least one BMI and/or mental health datapoint, then the participant was included in the sample, and any missing BMI and mental health data was imputed. Socioeconomic indicators (childhood and adulthood social class, and educational level) were available >98% of study participants. The imputation model also included several auxiliary variables from both cohorts (for example, birth weight, smoking during pregnancy and maternal breast feeding habits recorded in early childhood, and chronic illness, life satisfaction, partnership status, employment status, smoking habits and home ownership assessed in participants’ adulthood) to help strengthen the quality of imputed data. Lastly, we separately imputed data in each cohort, following which the two imputed datasets were appended together for further data analysis. All analyses presented are based on the imputed study sample (N=30 868). All analyses were run in Stata 15 (College Station, TX, USA).

## 4 Role of funding source

The funders had no role in the study design, data collection, data analysis, interpretation, the writing of the report or decisions on where to publish.

## 5 Results

Table 1 presents descriptive statistics for BMI and mental health, and the outcome variable (comorbidity of overweight and mental ill-health) across the five age sweeps. On average, mean BMI increased from 17.2kg/m^2^ at age 10 to 26.4kg/m^2^ at age 42. From age 16 onwards, the younger BCS70 cohort had higher mean BMI and higher proportions of participants being overweight compared to the NCDS58 cohort (for example, 19% vs. 33% at age 23 and 54% vs. 61% at age 42 being overweight in the NCDS58 and BCS70 cohorts respectively). Across adulthood, the younger BCS70 participants reported higher proportions of mental ill-health (for example 8.8 vs 14.6% at age 23 and 12.2 vs. 18% at age 42 in the NCDS58 and BCS70 cohorts respectively).

**Table 1:** Descriptive statistics of variables of interest in 30 868 participants from the 1958 National Child Development Study and the 1970 British Cohort Study. Numbers are means or percentages

	Age 11/10	Age 16	Age 23/26	Age 33/34	Age 42
	Mean or % (95% CI)	Mean or % (95% CI)	Mean or % (95% CI)	Mean or % (95% CI)	Mean or % (95% CI)
**Full study sample, N=30 868**					
Mental health^a^	2.8 (2.7-2.8)	2 (2.01-2.06)	1.53 (1.5-1.6)	1.4 (1.3-1.4)	1.7 (1.7-1.8)
High psych distress (%)	20 (19.2-20.2)	18 (17.6-18.6)	11.5 (11-11.9)	10.4 (10-10.8)	14.8 (14.3-15.3)
BMI (kg/m^2^)	17.2 (17.1-17.2)	20.9 (20.8-20.9)	23.2 (23.1-23.3)	25.5 (25.4-25.5)	26.4 (26.3-26.5)
Overweight or Obesity (%)	9 (8.7-9.4)	12.3 (11.9-12.8)	25.6 (25.1-26.2)	49 (48.3-49.6)	57.5 (56.8-58)
**Outcome of interest (%)**					
Healthy BMI & good mental health [↑BMI-MH↑]	73 (72.5-74)	71.8 (71.3-72.4)	66 (65.5-66.7)	46 (45.4-46.6)	36.6 (35.9-37.2)
OW/OB & good mental health [↑BMI-MH↓]	7 (6.9-7.5)	10 (9.5-10)	22.4 (21.9-22.9)	44 (42.9-44.2)	48.6 (47.9-49.2)
Healthy BMI & mental ill-health [↓BMI-MH↑]	17.8 (17.4-18.3)	15.7 (15.3-16)	8.3 (7.9-8.7)	5 (4.8-5.3)	6 (5.6-6.3)
OW/OB & mental ill-health [↓BMI-MH↓].	1.8 (1.6-1.9)	2.4 (2.1-2.5)	3.2 (2.9-3.4)	5.3 (5-5.6)	8.9 (8.4-9.2)
**NCDS58 only, N=16 464**					
Mental health^a^	3.26 (3.23-3.29)	1.9 (1.9-2.0)	1.3 (1.2-1.3)	1.04 (1.01-1.06)	1.5 (1.5-1.6)
High psych distress (%)	23.4 (22.7-24.1)	16.7 (16-17.3)	8.8 (8.3-9.2)	6.6 (6.1-7)	12.2 (11.7-12.8)
BMI (kg/m^2^)	17.5 (17.4-17.6)	20.7 (20.6-20.7)	22.7 (22.6-22.8)	25.03 (24.96-25.11)	25.9 (25.8-26.1)
Overweight or Obesity (%)	9.6 (9-10)	11 (10.4-11.5)	18.7 (18-19.4)	45 (44.1-45.9)	54.1 (53.2-54.9)
**Outcome of interest (%)**					
Healthy BMI & good mental health [↑BMI-MH↑]	69.2 (68.4-70)	74.2 (73.4-75)	74.2 (73.4-75)	51.3 (50.4-52.2)	40.3 (39.4-41.1)
OW/OB & good mental health [↑BMI-MH↓]	7.4 (7-7.8)	9.1 (8.6-9.6)	17 (16.3-17.6)	42 (41.2-43)	47.5 (46.6-48.4)
Healthy BMI & mental ill-health [↓BMI-MH↑]	21.3 (20.6-21.9)	14.9 (14.2-15.5)	7 (6.6-7.4)	3.7 (3.4-4)	5.6 (5.3-6.1)
OW/OB & mental ill-health [↓BMI-MH↓].	2.2 (1.9-2.4)	1.8 (1.6-2)	1.8 (1.5-2)	3 (2.6-3.2)	6.6 (6.1-6.9)
**BCS70 only, N=14 404**					
Mental health^a^	2.2 (2.1-2.3)	2.1 (2.1-2.2)	1.8 (1.8-1.9)	1.7 (1.70-1.8)	1.9 (1.90-2.0)
High psych distress (%)	15.4 (14.8-16)	20 (19.1-20.6)	14.6 (13.9-15.3)	14.7 (14-15.3)	18 (16.9-18.6)
BMI (kg/m^2^)	16.8 (16.8-16.9)	21.1 (21.1-21.2)	23.9 (23.8-23.9)	25.9 (25.8-26.1)	26.9 (26.8-27.1)
Overweight or Obesity (%)	8.5 (7.9-8.9)	14 (13.2-14.6)	33.5 (32.4-34.4)	53.4 (52.4-54.4)	61.3 (60.4-62.3)
**Outcome of interest (%)**					
Healthy BMI & good mental health [↑BMI-MH↑]	77.5 (76.8-78.2)	69.2 (68.4-70)	56.8 (55.7-57.8)	40 (39.1-41)	32.4 (31.4-33.3)
OW/OB & good mental health [↑BMI-MH↓]	7.0 (6.6-7.5)	11 (10.3-11.6)	28.7 (27.7-29.6)	45.3 (44.3-46.4)	50 (48.8-50.1)
Healthy BMI & mental ill-health [↓BMI-MH↑]	14 (13.4-14.6)	16.8 (16.1-17.6)	9.7 (9.2-10.4)	6.6 (6.1-7.1)	6.3 (5.7-6.8)
OW/OB & mental ill-health [↓BMI-MH↓].	1.4 (1.3-1.5)	3 (2.6-3.3)	4.8 (4.3-5.2)	8 (7.5-8.6)	11.5 (10.8-12.2)

a Mean values for mental health are based on symptoms of anxiety and depression measured by the Rutter internalising scale in childhood and the Malaise inventory in adulthood. Higher mean values indicate higher levels of anxiety and depression.

The proportion of participants with ↑BMI-MH↑ decreased progressively from 73% at age 10 to 37% at age 42. The largest increase was observed in the ↓BMI-MH↑ group (7% at age 10 to 49% at age 42). The ↓BMI-MH↓ group increased from 2% at age 10 to 9% at age 42. Across adulthood the proportion of participants with ↓BMI-MH↓ comorbidity was higher in the younger BCS70 cohort (for example, 2 vs. 5% at age 23 and 7 vs.12% at age 42 in NCDS58 and BCS70 cohorts respectively).

A higher proportion of BCS70 participants were in the most advantaged social class group (Table 2, 47 vs. 40% for adulthood social class and 27 vs. 23% for childhood social class in BCS70 and NCDS58 respectively).

**Table 2:** Distribution of 30 868 participants from the 1958 National Child Development Study and the 1970 British Cohort Study by sex and socioeconomic indicators of interest. Numbers are percentages (95% CI).

Covariates	Full study sample	NCDS58	BCS70
	N=30 868	N=16 464	N=14 404
**Sex**			
Males	51.4 (50.8-51.9)	51.3 (50.6-52.1)	51.4 (50.6-52.2)
Females	48.6 (48.1-49.2)	48.7 (47.9-49.4)	48.6 (47.7-49.4)
**Childhood social class**			
Professional & Managerial (Most advantaged)	24.6 (24.1-25.1)	22.7 (22.1-23.4)	26.7 (25.9-27.4)
Non-manual	10.4 (10-10.7)	10.2 (9.7-19.6)	10.6 (10.1-11.1)
Manual	42.2 (41.6-42.7)	42.9 (42.2-43.7)	41.2 (40.4-42.1)
Partly skilled & Unskilled	22.9 (22.4-23.4)	24.1 (23.5-24.8)	21.5 (20.8-22.1)
**Adulthood social class**			
Professional & Managerial (Most advantaged)	43.6 (42.8-44.3)	40.1 (39.2-41)	47.5 (46.4-48.7)
Non-manual	20.2 (19.6-20.8)	21.6 (20.8-22.4)	18.6 (17.7-19.4)
Manual	19.9 (19.3-20.5)	21 (20.2-21.8)	18.5 (17.6-19.4)
Partly skilled & Unskilled	16.4 (15.7-17)	17.3 (16.5-18)	15.4 (14.4-16.4)
**Highest educational level**			
None	12 (11.4-12.4)	13.3 (12.7-13.9)	10.3 (9.6-10.9)
Nvq1 level (Secondary education, lower grades)	10.7 (10.3-1.1)	12.8 (12.2-13.4)	8.4 (7.8-8.9)
Nvq2 level (Secondary education, higher grades)	32 (31.3-32.6)	34 (33.1-34.9)	29.7 (28.7-30.7)
Nvq3 level (School leaving certificate, A-levels)	14.2 (13.7-14.7)	13.9 (13.3-14.6)	14.5 (13.8-15.3)
Nvq4 level (Earning a degree)	21.8 (21.3-22.4)	13.8 (13.2-14.5)	31 (30-31.9)
Nvq5 level (University degree or diploma) (Most advantaged)	9.3 (8.9-9.6)	12.1 (11.5-12.7)	6.1 (5.7-6.6)

### 5.1 Inequalities in BMI and mental health over time

Supplemental Tables 2A and 2B show the variation in mean BMI and mental health by socioeconomic indicators at each age (i.e. the inequality gradient by social class and educational level, how it changes by age and between the two cohorts). In childhood, disadvantaged social class groups had higher levels of mental ill-health compared to the most advantaged. Similarly, differences in mean BMI were more visible at age 16 compared to age 10 and larger in BCS70 compared to NCDS58 (mean BMI 20.5 vs. 20.8kg/m^2^ in NCDS58 and 20.9 vs. 21.4kg/m^2^ in BCS70 for professional/managerial vs. partly skilled/unskilled respectively).

Differences in mean BMI and mental health between the most and least advantaged got wider, more consistent with age and were observed with all three socioeconomic indicators. The largest differences in mean BMI and mental health were observed with educational level (with a clear inverse gradient) in both cohorts. These differences were larger in the BCS70 cohort (for example, the mean difference in BMI for no education compared to having a degree was 1.8kg/m^2^ in NCDS58 and 2.5 kg/m^2^ in BCS70 at age 33, which increased to 2.3 kg/m^2^ in NCDS58 and 3kg/m^2^ in BCS70 at age 42). Mean differences in mental health between the most and least advantaged groups decreased marginally across adulthood.

### 5.2 Socioeconomic inequalities in risk for comorbidity between overweight and mental ill-health over time

**Figure 1:**
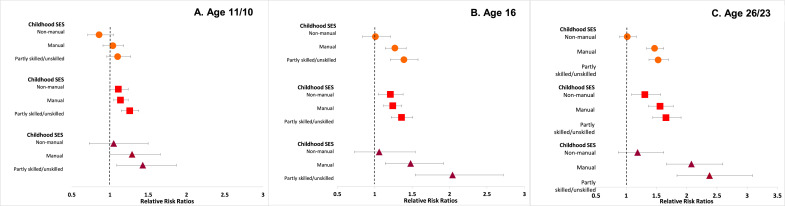
A, B, C. Relative risk ratios (RRR) for i. Overweight or obesity and good mental health, ii. Healthy BMI and mental ill-health and iii. Overweight or obesity and mental ill-health in 30 868 participants from the 1958 National Child Development Study and the 1970 British Cohort Study. Healthy BMI and good mental health is the reference category. Estimates are from multinomial logistic regression models. Note: Reference category for childhood social class is the professional/managerial group. Models are adjusted for sex and cohort.

**Figure 2:**
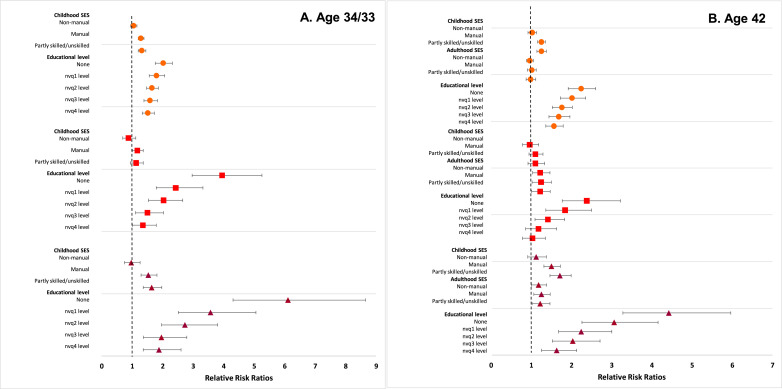
A & B. Relative risk ratios (RRR) for i. Overweight or obesity and good mental health, ii. Healthy BMI and mental ill-health and iii. Overweight or obesity and mental ill-health in 30 868 participants from the 1958 National Child Development Study and the 1970 British Cohort Study. Healthy BMI and good mental health is the reference category. Estimates are from multinomial logistic regression models. Note: Reference category for childhood social class and adulthood SES is the professional/managerial group. Reference category for educational level is NVQ 5 level (university degree). Models are adjusted for sex and cohort.

Supplemental Table 3 and Figure 1, Figure 2 display results from multinomial logistic regression examining risk for the different combinations of categorical BMI and mental health at each age (i.e. the associations between childhood and adulthood socioeconomic indicators and risk for comorbidity at each age). In general, the socioeconomically disadvantaged had independently higher risks for overweight and mental ill-health alone, and for comorbidity at all ages. In childhood, participants from more disadvantaged families had consistently higher relative risk ratios (RRR) for the three adverse groups. Compared to the most advantaged social class group (professional/managerial), the most disadvantaged group (partly skilled/unskilled) had the highest RRR for all three combinations of BMI and mental health (for example at age 16, RRR 1.39 [95% CI 1.21-1.58] for ↓BMI-MH↑, RRR 1.36 [1.22-1.51] for ↑BMI-MH↓, and RRR 2.04 [1.54-2.72] for ↓BMI-MH↓).

The associations between childhood social class and risk for the three adverse combinations of BMI and mental health were larger in effect size and observed consistently for both manual and partly skilled/unskilled groups at age 23 (compared to associations observed in childhood).

There was a clear inverse gradient between educational level and risk for the three adverse combinations of BMI and mental health at ages 34 and 42. Compared to those with a university degree, all other educational categories had higher risk for the three adverse combinations of BMI and mental health, with the risk increasing as educational levels decreased, even after mutual adjustment for other socioeconomic indicators.

We observed a very similar pattern in increased risk for the three adverse groups when multinomial logistic models were run in complete case data (N=30,868) with the same inverse gradient with educational level as described above (Supplemental Table 4). However, the observed RRRs were smaller in size, as would be expected given that attrition in the studies is predicted by both socio-economic disadvantage and poorer health; thus leading to underestimation of their association in complete cases.

A disadvantaged childhood social class was independently associated with increased risk of ↓BMI-MH↓ comorbidity in adulthood (adjusted RRR 2.38 [1.84-3.09] at age 23 and 1.71 [1.46-1.99] at age 42 for the partly skilled/unskilled group).

Supplemental Table 5 displays results from multinomial logistic regression examining risk for the different combinations of categorical BMI and mental health at each age stratified by cohort. Compared to NCDS58 participants, BCS70 participants had an increased risk for all three adverse combinations of BMI and mental health with the largest risks for ↓BMI-MH↓ comorbidity (Supplemental Table 5, RRR 3.69 [3.13-4.35] at age 23 and 3.95 [3.47-4.5] at age 34).

**Figure 3:**
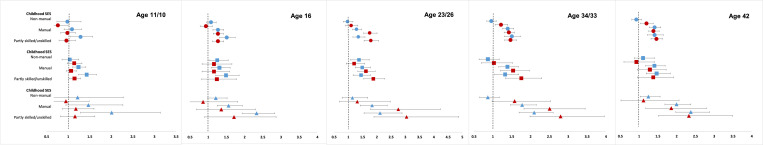
Relative risk ratios (RRR) between childhood social class and risk for i. Overweight or obesity and good mental health, ii. Healthy BMI and mental ill-health and iii. Overweight or obesity and mental ill-health in 30,868 participants from the 1958 National Child Development Study and the 1970 British Cohort Study. Healthy BMI and good mental health is the reference category. Estimates are from multinomial logistic regression models. Note: Reference category for childhood social class is the professional/managerial group. Models are adjusted for sex and cohort.

Figure 3 and Supplemental Table 6 display results from multinomial regression modelling examining risk of comorbidity with only childhood social class at all ages and stratified by cohort (i.e. the independent association between childhood social class and risk for comorbidity at each age). At all ages, a disadvantaged childhood social class was associated with increased risk for all adverse groups, with RRR getting progressively larger with age. The largest risk was observed for ↓BMI-MH↓ comorbidity at all ages and in both cohorts (for example, RRR 1.71 [1.12-2.61] and 2.33 [1.63-3.32] at age 16 and RRR 2.1 [1.70-2.60] and 2.33 [1.87-2.89] at age 42 for the partly skilled/unskilled group in NCDS58 and BCS70 respectively). While BCS70 participants had higher RRR for ↓BMI-MH↓ comorbidity in childhood compared to NCDS70, the reverse was observed at ages 23 and 34. However, confidence intervals for cohort-specific RRR were largely overlapping.

### 5.3 Differences in socioeconomic inequalities in comorbidity by cohort and sex

Risk for the three adverse combinations of comorbidity between the most and least advantaged differed between the two cohorts at all ages except age 16 (tests for interaction; age 10, p<0.05, ages 23, 34 and 42, p<0.001) (Supplemental Table 5 and Supplemental Figure 2). At age 10, participants in the younger BCS70 cohort had higher risk for all three adverse combinations compared to the NCDS58 cohort (Supplemental Figure 2A). At ages 34/33 and 42, NCDS58 participants with no education had higher risk of ↑BMI-MH↓ and ↓BMI-MH↓ comorbidity compared to BCS70 participants (Supplemental Figures 2C and 2D).

Interactions tests between sex and socioeconomic indictors did not indicate that the socioeconomic patterns observed differed between in males and females (this should be interpreted with caution as lack of statistically significant interactions tests doesn't necessarily imply no differences in socioeconomic inequalities between sexes).

## 6 Discussion

This prospective observational cohort study found 1. Consistent associations between socioeconomic disadvantage and increased risk of co-morbid overweight and mental ill-health from childhood into mid-adulthood, 2. While socioeconomic disadvantage was associated with increased risks of either overweight or mental ill-health in childhood and adulthood, the greatest inequalities were observed for their comorbidity. 3. Greater levels of socioeconomic disadvantage were associated with increasing risks of comorbidity, with inverse gradients observed with social class and educational level, 4. Disadvantaged childhood social class was associated with increased risk of comorbidity in adulthood even after accounting for adulthood socioeconomic circumstances. 5. Differences by cohort and sex in the extent of socioeconomic inequalities in risk for comorbidity were limited.

This study benefits from a large sample size drawn from two nationally representative birth cohorts with multiple BMI and mental health measurements. Like most longitudinal studies, both cohorts suffer from attrition (66 and 57% of the NCDS58 and BCS70 cohorts respectively, were maintained up to age 42) but this was addressed using the robust multiple imputation technique. While imputation relies on the MAR assumption which is not empirically verifiable [38], we increased the plausibility of the MAR assumption by including a varied and rich set of auxiliary variables in the imputation model [39]. These auxiliary variables contribute information which helps predict missing data with greater precision and minimising non-random variation in the values [40]. Further, longitudinal data on BMI and mental health are powerful predictors of missing data which substantially strengthened the imputation model and reliability of the imputed data. Nonetheless, attrition in longitudinal studies is often higher in socioeconomically disadvantaged groups and those with obesity and other health conditions, which has showed a reduction in magnitude of observed inequalities [41]. While BMI was self-reported at some ages in both cohorts, studies indicate that self-reported measurements do not substantially bias estimates [42]. Extent of inequalities for mental health in childhood could be overestimated, as previous studies show greater socioeconomic inequalities in mental health when using parent- compared to individual-reported data [43]. This study benefits from the use of childhood and adulthood socioeconomic indicators which help understand the independent contribution of these markers on comorbidity risk across time. Inferences on cross-cohort comparisons are strengthened by using harmonised socioeconomic indicators, BMI and mental health measures.

To our knowledge no study has examined socioeconomic inequalities in risk of comorbid overweight/obesity and mental ill-health in the same individuals over time. However, socioeconomic inequalities in BMI and mental health are separately well established across the lifecourse and these inequalities have increased in younger generations [8,15,17]. Being socioeconomically disadvantaged is not only a common risk factor for both mental ill-health and obesity, it is linked to other factors on the pathway to either condition. For example, living in disadvantaged neighbourhoods is often associated with decreased access to recreational areas, green spaces and healthier food [[44], [45], [46]]. The association between obesity and mental ill-health is bidirectional (obesity leads to mental ill-health and vice versa [47]) but both conditions can be a result of common risk factors more likely to impact disadvantaged groups like increased stress levels, health behaviours (poorer eating habits, inadequate exercise), social stigma, higher unemployment etc. Socioeconomically disadvantaged individuals are more likely to experience ongoing and continuing stressors which impact their health across the lifecourse leading to an accumulative effect which perpetuates health inequalities [8]. Studies have indicated common biological pathways that might explain the link between obesity and depression, particularly the activation of the hypothalamic–pituitary–adrenal (HPA) axis [48]. Exposure to early life stress and chronic stress over time can lead to hyperactivation of the HPA-axis resulting in high levels of cortisol associated with increased risk for both depression and obesity [48].

Prevalence of obesity and mental ill-health were higher in adulthood in the younger BCS70 cohort, yet cohort differences in socioeconomic inequalities in comorbidity were limited (with some indication of greater inequalities in the older NCDS58 cohort). One reason could be the shift in distribution of socioeconomic position, with the younger BCS cohort having higher number of individuals in the more socioeconomically advantaged groups. It is also possible that socioeconomic inequalities in comorbidity might manifest differently at older ages in the two cohorts which can be examined in the future. Socioeconomically disadvantaged women are more vulnerable to obesity and depression compared to men, yet our study found no sex differences in socioeconomic inequalities in comorbidity [[49], [50], [51], [52]]. However, we caution that lack of statistically significant interactions tests doesn't necessarily imply that there are no differences in socioeconomic inequalities in comorbidity between the two cohorts or sexes, rather they were not observed in this study population.

Health inequalities have not only persisted but widened in recent decades despite significant policies developed to tackle them. The theory of syndemics suggests that diseases cluster together producing greater impacts on health (synergism) at the population- and individual-level and is one of the mechanisms by which health inequalities develop and perpetuate in the socioeconomically disadvantaged [53,54]. Hence, any model developed to reduce inequalities associated with comorbidity will have to address not only obesity and mental ill-health but also associated risk factors and conditions that drive them while incorporating macro- and individual-level factors. A syndemic approach in reducing inequalities should include targeting common root causes of comorbid conditions at neighbourhood-level (like promoting healthier lifestyles like better and easy access to recreational spaces, affordable gyms, and healthy food options, integrated healthcare services, while accounting for local population factors like unemployment and ethnic diversity for example). Additionally, such services should be more accessible to disadvantaged populations (that is while universal in nature, they must be proportional with increased targeting towards the more disadvantaged like unemployed, low income, lone parents or ethnic-minority individuals without creating any stigma [55]). Most public health policies addressing health inequalities target ‘’downstream’’ factors such as lifestyle behaviours [56]. However, if we are to truly reduce health inequalities with a long lasting impact, we also have to address ‘’upstream’’ factors, i.e. the structural policies and drivers which determine the context within which downstream factors take place (ultimately influencing lifestyle behaviours for example). Examples of upstream factors could include market regulation of unhealthy food products, reducing sugar and salt content in processed foods, making healthier food options more affordable and policies developed to ensure greater income equality [56]. Lastly, public health policies to reduce inequalities must have a lifecourse dimension targeting all ages as conditions like obesity and mental ill-health are more likely to occur separately and together in socioeconomically disadvantaged groups across the lifecourse as shown in this study [57]. An interesting (and unexamined) extension of this study would be to analyse socioeconomic differences in risk for comorbidity in those individuals that experience a change in SEP between childhood and adulthood (low SEP to high SEP and vice versa). Lastly, in light of secular trends in health, and in widening childhood and adulthood socioeconomic inequalities, these findings have significant implications for more recent cohorts who face much higher levels of morbidity (including obesity and mental ill-health) as they age. Our study highlights the long reaching consequences of disadvantaged childhood circumstances on health across the lifecourse, regardless of adulthood socioeconomic position.

In conclusion, this large study using two national birth cohorts found robust evidence for socioeconomic inequalities in risk of being overweight, having mental ill-health and comorbidity between the two conditions in individuals followed from childhood to mid-adulthood. Associations were consistent with both childhood and adulthood socioeconomic indicators and got progressively larger over time, with the most disadvantaged individuals experiencing the greatest risk. Lastly, socioeconomic disadvantage in childhood was independently associated with increased risk of comorbidity in mid-adulthood.

## Declaration of Competing Interest

### Funding

This research was supported by grants from the Wellcome Trust (ISSF3/ H17RCO/NG1) and Medical Research Council (MRC) [MC_UU_00019/3].

### Conflicts of Interest

None.

### Availability of data and material (data transparency)

Anonymised data from the 1958 National Survey of Health and Development (NCDS) and the 1970 British Cohort Study (BCS) can be obtained free of charge from the UK Data Service (for more information: https://cls.ucl.ac.uk/data-access-training/).

### Code availability (software application or custom code)

Available upon request.

## Authors' contributions

AK and PP conceived the research idea. AK conducted the data preparation and data analysis. Both authors were involved in the research design, interpretation of results and writing the manuscript. Both authors read and approved the final submitted manuscript.

## Appendix Supplementary materials

Image, application 1

## Footnotes

Supplementary material associated with this article can be found, in the online version, at doi:10.1016/j.lanepe.2021.100106.
