# Supplementary material for: Socioeconomic inequalities in co-morbidity of overweight, obesity and mental ill-health from adolescence to mid-adulthood in two national birth cohort studies
Source: Lancet Reg Health Eur. 2021 Apr 30;6:100106. doi: 10.1016/j.lanepe.2021.100106 (PMC8291042; doi:10.1016/j.lanepe.2021.100106)

**Supplemental Figure 1. A flow chart explaining how the study sample was conceived**

**NCDS58 BCS70**

**Birth sample, N=17,416 Birth sample, N=16,571 Supplemental Table 1.** Questions that comprise the Rutter internalising scale and the Malaise inventory used to assess psychological distress (symptoms of depression and anxiety)

**Age 10,** N=14,874

**Age 16,** N=11,621

**Age 26,** N=9,003

**Age 34,** N=9,656

**Age 42,** N=9,841

**Final N=30,868**

Min 1 BMI *or* Mental health datapoint, N=14,404

Min 1 BMI *or* Mental health datapoint, N=16,464

**Age 11,** N=15,337

**Age 16,** N=14,647

**Age 23,** N=12,537

**Age 33,** N=11,407

**Age 42,** N=11,419

The first five items listed are common to both scales

|  | **Rutter Internalising scale** | **Malaise inventory** |
| --- | --- | --- |
|  |  |  |
| 1 | Often appears miserable, unhappy, tearful or distressed. | Do you often feel miserable or depressed? |
| 2 | Irritable. Is quick to fly off the handle. | Do people annoy and irritate you? |
| 3 | Often worried, worries about many things. | Do you often get worried about things? |
| 4 | Tends to be fearful or afraid of new things or new situations. | Do you suddenly become scared for no good reason? |
| 5 | Has twitches, mannerisms or tics of the face or body. | Have you at times had a twitching of the face, head or shoulders? |
| 6 |  | Do you feel tired? |
| 7 |  | Do you ever get in a violent rage? |
| 8 |  | Are you keyed up and jittery? |
| 9 |  | Does your heart race like mad? |

**Supplemental Table 2A. Distributions of BMI and mental health (psychological distress) in 30 868 participants from the 1958 National Child Development Study and the 1970 British Cohort Study by socioeconomic indicators in childhood**

|  | **Age 11/10** | | **Age 16** | |
| --- | --- | --- | --- | --- |
|  | **BMI (kg/m^2^)** | **Mental health^a^** | **BMI (kg/m^2^)** | **Mental health^a^** |
|  | Mean (95% CI) | Mean (95% CI) | Mean (95% CI) | Mean (95% CI) |
| **NCDS58 only, N=16,464** |  |  |  |  |
| **Childhood social class** |  |  |  |  |
| Professional & Managerial | 17.6 (17.5-17.6) | 3.1 (3-3.2) | 20.5 (20.4-20.6) | 1.8 (1.8-1.9) |
| Non-manual | 17.4 (17.2-17.5) | 3.4 (3.3-3.5) | 20.5 (20.4-20.7) | 2.0 (1.9-2.1) |
| Manual | 17.5 (17.4-17.6) | 3.3 (3.2-3.4) | 20.7 (20.6-20.8) | 2.0 (1.9-2) |
| Partly skilled & Unskilled | 17.4 (17.3-17.5) | 3.3 (3.2-3.4) | 20.8 (20.7-20.9) | 2.0 (1.9-2.1) |
|  |  |  |  |  |
| **BCS70 only, N=14,404** |  |  |  |  |
| **Childhood social class** |  |  |  |  |
| Professional & Managerial | 16.8 (16.7-16.9) | 2.0 (1.9-2.1) | 20.9 (20.8-21.1) | 1.9 (1.8-2) |
| Non-manual | 16.8 (16.7-17) | 2.1 (2-2.2) | 20.9 (20.8-21.1) | 2.1 (1.9-2.2) |
| Manual | 16.9 (16.8-17) | 2.3 (2.2-2.4) | 21.2 (21.1-21.3) | 2.2 (2.1-2.3) |
| Partly skilled & Unskilled | 16.9 (16.8-17) | 2.5 (2.4-2.6) | 21.4 (21.2-21.5) | 2.3 (2.2-2.4) |
|  |  |  |  |  |

^a^Mean values for mental health are based on symptoms of anxiety and depression measured by the Rutter internalising scale in childhood and the Malaise inventory assessed in adulthood. Higher mean values indicate higher levels of anxiety and depression.

**Supplemental Table 2B. Distributions of BMI and mental health (psychological distress) in 30 868 participants from the 1958 National Child Development Study and the 1970 British Cohort Study by socioeconomic indicators in adulthood**

|  | **Age 23/26** | | **Age 34/33** | | **Age 42** | |
| --- | --- | --- | --- | --- | --- | --- |
|  | **BMI (kg/m^2^)** | **Mental^a^ health** | **BMI (kg/m^2^)** | **Mental^a^ health** | **BMI (kg/m^2^)** | **Mental health^a^** |
|  | Mean (95% CI) | Mean (95% CI) | Mean (95% CI) | Mean (95%CI) | Mean (95% CI) | Mean (95% CI) |
| ***NCDS58 only, N=16 464*** |  |  |  |  |  |  |
| **Childhood social class** |  |  |  |  |  |  |
| Professional & Managerial (most advantaged) | 22.2 (22.1-22.3) | 1.0 (0.9-1.1) | 24.4 (24.2-24.5) | 0.9 (0.8-0.9) | 25.2 (25.1-25.4) | 1.4 (1.4-1.5) |
| Non-manual | 22.3 (22.1-22.4) | 1.1 (1-1.2) | 24.8 (24.6-25.1) | 1.0 (0.9-1) | 25.6 (25.4-25.9) | 1.4 (1.3-1.5) |
| Manual | 22.9 (22.8-23) | 1.3 (1.3-1.4) | 25.3 (25.2-25.4) | 1.1 (1-1.1) | 26.2 (26.1-26.3) | 1.6 (1.5-1.6) |
| Partly skilled & Unskilled | 22.9 (22.8-23.1) | 1.5 (1.4-1.5) | 25.3 (25.1-25.5) | 1.2 (1.1-1.2) | 26.3 (26.2-26.5) | 1.7 (1.6-1.8) |
| **Adulthood social class** |  |  |  |  |  |  |
| Professional & Managerial (most advantaged) | 22.4 (22.4-22.5) | 1.0 (0.9-1.1) | 24.9 (24.7-25) | 0.8 (0.8-0.9) | 25.8 (25.7-26) | 1.3 (1.3-1.4) |
| Non-manual | 22.2 (22.1-22.4) | 1.5 (1.4-1.6) | 24.5 (24.3-24.7) | 1.2 (1.1-1.3) | 25.5 (25.3-25.6) | 1.8 (1.7-1.9) |
| Manual | 23.3 (23.1-23.4) | 1.2 (1.1-1.3) | 25.6 (25.4-25.8) | 1.0 (0.9-1.1) | 26.5 (26.3-26.7) | 1.5 (1.4-1.6) |
| Partly skilled & Unskilled | 23.0 (22.8-23.2) | 1.7 (1.6-1.8) | 25.4 (25.1-25.6) | 1.5 (1.4-1.6) | 26.2 (25.9-26.4) | 1.8 (1.7-1.9) |
| **Highest educational level** |  |  |  |  |  |  |
| None | 23.5 (23.3-23.7) | 2.2 (2.1-2.3) | 25.8 (25.5-26) | 1.7 (1.6-1.8) | 27.0 (26.6-27.3) | 2.2 (2.1-2.3) |
| Nvq1 level | 23.0 (22.8-23.2) | 1.6 (1.5-1.7) | 25.5 (25.2-25.7) | 1.3 (1.2-1.4) | 26.5 (26.2-26.8) | 1.7 (1.6-1.8) |
| Nvq2 level | 22.7 (22.6-22.8) | 1.2 (1.1-1.3) | 25.0 (24.9-25.2) | 1 (0.9-1.1) | 25.9 (25.8-26.1) | 1.5 (1.4-1.6) |
| Nvq3 level | 22.5 (22.4-22.7) | 0.9 (0.8-0.9) | 25.0 (24.8-25.2) | 0.8 (0.7-0.9) | 25.8 (25.6-26) | 1.4 (1.3-1.5) |
| Nvq4 level | 22.4 (22.2-22.6) | 0.9 (0.8-1) | 24.9 (24.7-25.1) | 0.8 (0.7-0.8) | 25.9 (25.7-26.1) | 1.3 (1.2-1.4) |
| Nvq5 level (most advantaged) | 21.8 (21.6-22) | 0.8 (0.7-0.9) | 24.0 (23.8-24.3) | 0.7 (0.6-0.8) | 24.7 (24.5-25) | 1.3 (1.2-1.4) |
| ***BCS70 only, N=14 404*** |  |  |  |  |  |  |
| **Childhood social class** |  |  |  |  |  |  |
| Professional & Managerial (most advantaged) | 23.6 (23.4-23.7) | 1.6 (1.5-1.7) | 25.4 (25.2-25.5) | 1.6 (1.5-1.6) | 26.3 (26.1-26.4) | 1.7 (1.6-1.8) |
| Non-manual | 23.4 (23.2-23.6) | 1.8 (1.6-1.9) | 25.3 (25.1-25.6) | 1.6 (1.5-1.7) | 26.2 (25.9-26.5) | 1.9 (1.8-2) |
| Manual | 24.0 (23.9-24.1) | 1.9 (1.9-2) | 26.2 (26.1-26.3) | 1.8 (1.8-1.9) | 27.4 (27.2-27.5) | 2.0 (1.9-2.1) |
| Partly skilled & Unskilled | 24.2 (24-24.4) | 2.0 (1.9-2.1) | 26.5 (26.3-26.8) | 1.9 (1.8-2) | 27.6 (27.3-27.8) | 2.2 (2.1-2.3) |
| **Adulthood social class** |  |  |  |  |  |  |
| Professional & Managerial (most advantaged) | 23.6 (23.5-23.7) | 1.6 (1.5-1.7) | 25.6 (25.4-25.7) | 1.5 (1.5-1.6) | 26.6 (26.4-26.7) | 1.7 (1.6-1.8) |
| Non-manual | 23.8 (23.6-24) | 1.9 (1.8-2.1) | 25.9 (25.6-26.1) | 1.9 (1.8-2) | 27.0 (26.8-27.3) | 2.1 (2-2.3) |
| Manual | 24.4 (24.1-24.7) | 1.8 (1.7-1.9) | 26.7 (26.5-27) | 1.8 (1.6-1.9) | 27.8 (27.5-28.1) | 2.0 (1.9-2.1) |
| Partly skilled & Unskilled | 24.1 (23.9-24.3) | 2.3 (2.1-2.5) | 26.3 (26-26.6) | 2.0 (1.9-2.2) | 27.3 (27-27.7) | 2.3 (2.2-2.5) |
|  |  |  |  |  |  |  |
| **Highest educational level** |  |  |  |  |  |  |
| None | 24.2 (23.9-24.6) | 2.4 (2.2-2.5) | 26.7 (26.4-27.0) | 2.2 (2.1-2.3) | 27.8 (27.5-28.2) | 2.4 (2.2-2.5) |
| Nvq1 level | 24.0 (23.7-24.3) | 2.3 (2.1-2.5) | 26.4 (26-26.7) | 2.0 (1.8-2.1) | 27.7 (27.2-28.1) | 2.4 (2.2-2.6) |
| Nvq2 level | 24.0 (23.8-24.2) | 1.9 (1.8-2.0) | 26.1 (25.9-26.3) | 1.9 (1.7-1.9) | 27.3 (27-27.5) | 2.1 (2-2.2) |
| Nvq3 level | 23.8 (23.6-24.1) | 1.7 (1.6-1.8) | 26.1 (25.8-26.3) | 1.6 (1.5-1.7) | 27.1 (26.8-27.5) | 1.8 (1.7-1.9) |
| Nvq4 level | 23.8 (23.6-23.9) | 1.6 (1.5-1.7) | 25.7 (25.5-25.9) | 1.5 (1.4-1.6) | 26.6 (26.4-26.8) | 1.7 (1.6-1.8) |
| Nvq5 level (most advantaged) | 22.8 (22.6-23.1) | 1.4 (1.2-1.6) | 24.2 (23.9-24.6) | 1.3 (1.2-1.5) | 24.8 (24.4-25.2) | 1.6 (1.4-1.7) |

^a^Mean values for mental health are based on symptoms of anxiety and depression measured by the Rutter internalising scale in childhood and the Malaise inventory assessed in adulthood. Higher mean values indicate higher levels of anxiety and depression.

**Supplemental Table 3. Relative risk ratios (RRR) for i. overweight or obesity and good mental health, ii. Healthy BMI and mental ill-health and iii. Overweight or obesity and mental ill-health in 30 868 participants from the 1958 National Child Development Study and the 1970 British Cohort Study (pooled cohort analysis). Healthy BMI and normal mental health is the reference category.**

|  | **Age 11/10** | | **Age 16** | | **Age 23/26** | | **Age 34/33** | | **Age 42** | |
| --- | --- | --- | --- | --- | --- | --- | --- | --- | --- | --- |
|  | **RRR** | **95% CI** | **RRR** | **95% CI** | **RRR** | **95% CI** | **RRR** | **95% CI** | **RRR** | **95% CI** |
|  |  |  |  |  |  |  |  |  |  |  |
| ***Healthy BMI & good MH*** | **Ref** |  | **Ref** |  | **Ref** |  | **Ref** |  | **Ref** |  |
|  |  |  |  |  |  |  |  |  |  |  |
| ***Overweight/Obese & good MH*** |  |  |  |  |  |  |  |  |  |  |
| **Male** | **1** |  | **1** |  | **1** |  | **1** |  | **1** |  |
| **Female** | **1.49** | **[1.35,1.64]** | **1.31** | **[1.20,1.43]** | 0.6 | [0.55,0.64] | 0.5 | [0.47,0.53] | 0.5 | [0.47,0.53] |
| **Childhood social class** |  |  |  |  |  |  |  |  |  |  |
| Professional & managerial | **1** |  |  |  | **1** |  | **1** |  | **1** |  |
| Non-manual | 0.86 | [0.71,1.05] | 1.01 | [0.84,1.21] | 1.02 | [0.89,1.17] | 1.05 | [0.95,1.16] | 1.02 | [0.92,1.13] |
| Manual | 1.04 | [0.91,1.18] | **1.27** | **[1.14,1.42]** | **1.47** | **[1.34,1.62]** | **1.29** | **[1.20,1.39]** | **1.25** | **[1.16,1.35]** |
| Partly & unskilled | 1.1 | [0.96,1.27] | **1.39** | **[1.21,1.58]** | **1.53** | **[1.38,1.70]** | **1.32** | **[1.20,1.45]** | **1.25** | **[1.14,1.37]** |
| **Adulthood social class** |  |  |  |  |  |  |  |  |  |  |
| Professional & managerial |  |  |  |  |  |  |  |  | **1** |  |
| Non-manual |  |  |  |  |  |  |  |  | 0.96 | [0.88,1.05] |
| Manual |  |  |  |  |  |  |  |  | 1.01 | [0.90,1.12] |
| Partly & unskilled |  |  |  |  |  |  |  |  | 0.98 | [0.87,1.11] |
| **Educational Level** |  |  |  |  |  |  |  |  |  |  |
| None |  |  |  |  |  |  | **2.02** | **[1.76,2.32]** | **2.24** | **[1.92,2.60]** |
| nvq1 level |  |  |  |  |  |  | **1.8** | **[1.56,2.07]** | **2.01** | **[1.72,2.35]** |
| nvq2 level |  |  |  |  |  |  | **1.65** | **[1.47,1.86]** | **1.76** | **[1.52,2.02]** |
| nvq3 level |  |  |  |  |  |  | **1.59** | **[1.39,1.83]** | **1.68** | **[1.44,1.96]** |
| nvq4 level |  |  |  |  |  |  | **1.52** | **[1.33,1.73]** | **1.56** | **[1.36,1.79]** |
| nvq5 level |  |  |  |  |  |  | **1** |  | **1** |  |
| **Cohort** |  |  |  |  |  |  |  |  |  |  |
| NCDS58 | **1** |  | **1** |  | **1** |  | **1** |  | **1** |  |
| BCS70 | **0.86** | **[0.78,0.94]** | **1.30** | **[1.18,1.43]** | **2.27** | **[2.1,2.45]** | **1.4** | **[1.31,1.5]** | **1.33** | **[1.25,1.41]** |
|  |  |  |  |  |  |  |  |  |  |  |
| ***Healthy BMI & mental ill-health*** |  |  |  |  |  |  |  |  |  |  |
| **Male** | **1** |  | **1** |  | **1** |  | **1** |  | **1** |  |
| **Female** | **1.03** | **[0.97,1.10]** | **1.4** | **[1.30,1.50]** | **2.47** | **[2.22,2.74]** | **1.61** | **[1.42,1.83]** | **1.42** | **[1.26,1.61]** |
| **Childhood social class** |  |  |  |  |  |  |  |  |  |  |
| Professional & managerial | **1** |  | **1** |  |  |  | **1** |  | **1** |  |
| Non-manual | 1.11 | [0.99,1.24] | **1.21** | **[1.05,1.38]** | **1.31** | **[1.09,1.57]** | 0.88 | [0.69,1.12] | 0.96 | [0.78,1.18] |
| Manual | **1.14** | **[1.05,1.24]** | **1.24** | **[1.12,1.36]** | **1.56** | **[1.37,1.78]** | **1.17** | **[1.01,1.36]** | 1.1 | [0.94,1.29] |
| Partly & unskilled | **1.26** | **[1.15,1.38]** | **1.36** | **[1.22,1.51]** | **1.66** | **[1.44,1.91]** | 1.13 | [0.94,1.36] | 1.1 | [0.92,1.33] |
| **Adulthood social class** |  |  |  |  |  |  |  |  |  |  |
| Professional & managerial |  |  |  |  |  |  |  |  | **1** |  |
| Non-manual |  |  |  |  |  |  |  |  | **1.22** | **[1.03,1.46]** |
| Manual |  |  |  |  |  |  |  |  | **1.24** | **[1.02,1.50]** |
| Partly & unskilled |  |  |  |  |  |  |  |  | **1.22** | **[1.01,1.47]** |
| **Educational Level** |  |  |  |  |  |  |  |  |  |  |
| None |  |  |  |  |  |  | **3.95** | **[2.97,5.26]** | **2.38** | **[1.77,3.22]** |
| nvq1 level |  |  |  |  |  |  | **2.43** | **[1.79,3.32]** | **1.84** | **[1.36,2.50]** |
| nvq2 level |  |  |  |  |  |  | **2.03** | **[1.54,2.66]** | **1.41** | **[1.09,1.83]** |
| nvq3 level |  |  |  |  |  |  | **1.5** | **[1.11,2.03]** | 1.18 | [0.86,1.63] |
| nvq4 level |  |  |  |  |  |  | **1.35** | **[1.01,1.79]** | 1.03 | [0.78,1.35] |
| nvq5 level |  |  |  |  |  |  | **1** |  | **1** |  |
| **Cohort** |  |  |  |  |  |  |  |  |  |  |
| NCDS58 | **1** |  | **1** |  | **1** |  | **1** |  | **1** |  |
| BCS70 | **0.59** | **[0.56,0.63]** | **1.23** | **[1.15,1.32]** | **1.86** | **[1.69,2.05]** | **2.53** | **[2.22,2.89]** | **1.5** | **[1.32,1.71]** |
|  |  |  |  |  |  |  |  |  |  |  |
| ***Overweight/obese & mental ill-health*** |  |  |  |  |  |  |  |  |  |  |
| **Male** | **1** |  | **1** |  | **1** |  | **1** |  | **1** |  |
| **Female** | **1.31** | **[1.09,1.57]** | **1.61** | **[1.33,1.96]** | **1.47** | **[1.25,1.72]** | 1 | [0.89,1.13] | **0.82** | **[0.74,0.91]** |
| **Childhood social class** |  |  |  |  |  |  |  |  |  |  |
| Professional & managerial | **1** |  | **1** |  | **1** |  | **1** |  | **1** |  |
| Non-manual | 1.05 | [0.73,1.50] | 1.06 | [0.73,1.54] | 1.19 | [0.87,1.62] | 0.97 | [0.75,1.26] | 1.12 | [0.91,1.37] |
| Manual | **1.29** | **[1.00,1.66]** | **1.48** | **[1.14,1.92]** | **2.08** | **[1.67,2.60]** | **1.53** | **[1.29,1.81]** | **1.5** | **[1.31,1.72]** |
| Partly & unskilled | **1.43** | **[1.09,1.87]** | **2.04** | **[1.54,2.72]** | **2.38** | **[1.84,3.09]** | **1.64** | **[1.36,1.97]** | **1.71** | **[1.46,1.99]** |
| **Adulthood social class** |  |  |  |  |  |  |  |  |  |  |
| Professional & managerial |  |  |  |  |  |  |  |  | **1** |  |
| Non-manual |  |  |  |  |  |  |  |  | **1.18** | **[1.00,1.38]** |
| Manual |  |  |  |  |  |  |  |  | **1.25** | **[1.06,1.47]** |
| Partly & unskilled |  |  |  |  |  |  |  |  | **1.22** | **[1.02,1.46]** |
| **Educational Level** |  |  |  |  |  |  |  |  |  |  |
| None |  |  |  |  |  |  | **6.11** | **[4.31,8.65]** | **4.42** | **[3.28,5.96]** |
| nvq1 level |  |  |  |  |  |  | **3.57** | **[2.52,5.06]** | **3.06** | **[2.26,4.15]** |
| nvq2 level |  |  |  |  |  |  | **2.73** | **[1.96,3.80]** | **2.24** | **[1.68,3.00]** |
| nvq3 level |  |  |  |  |  |  | **1.96** | **[1.37,2.79]** | **2.03** | **[1.52,2.71]** |
| nvq4 level |  |  |  |  |  |  | **1.88** | **[1.36,2.60]** | **1.63** | **[1.25,2.13]** |
| nvq5 level |  |  |  |  |  |  | **1** |  | **1** |  |
| **Cohort** |  |  |  |  |  |  |  |  |  |  |
| NCDS58 | **1** |  | **1** |  | **1** |  | **1** |  | **1** |  |
| BCS70 | **0.58** | **[0.48,0.71]** | **1.83** | **[1.52,2.19]** | **3.69** | **[3.13,4.35]** | **3.95** | **[3.47,4.5]** | **2.42** | **[2.16,2.72]** |

**Supplemental Table 4.** **Relative risk ratios (RRR) for i. overweight or obesity and good mental health, ii. Healthy BMI and mental ill-health and iii. Overweight or obesity and mental ill-health in 30 868 participants (complete cases) from the 1958 National Child Development Study and the 1970 British Cohort Study (pooled cohort analysis). Healthy BMI and normal mental health is the reference category.**

|  | **Age 11/10** | | **Age 16** | | **Age 23/26** | | **Age 34/33** | | **Age 42** | |
| --- | --- | --- | --- | --- | --- | --- | --- | --- | --- | --- |
|  | **RRR** | **95% CI** | **RRR** | **95% CI** | **RRR** | **95% CI** | **RRR** | **95% CI** | **RRR** | **95% CI** |
|  |  |  |  |  |  |  |  |  |  |  |
| ***Healthy BMI & good MH*** | **Ref** |  | **Ref** |  | **Ref** |  | **Ref** |  | **Ref** |  |
| ***Overweight/Obese & good MH*** |  |  |  |  |  |  |  |  |  |  |
| Male | 1 | [1.00,1.00] | 1 | [1.00,1.00] | 1 | [1.00,1.00] | 1 | [1.00,1.00] | 1 | [1.00,1.00] |
| Female | 1.46 | [1.35,1.59] | 1.3 | [1.22,1.38] | 0.61 | [0.58,0.64] | 0.51 | [0.49,0.53] | 0.51 | [0.49,0.54] |
| **Cohort** |  |  |  |  |  |  |  |  |  |  |
| NCDS58 | 1 | [1.00,1.00] | 1 | [1.00,1.00] | 1 | [1.00,1.00] | 1 | [1.00,1.00] | 1 | [1.00,1.00] |
| BCS70 | 0.86 | [0.79,0.93] | 1.29 | [1.21,1.37] | 2.16 | [2.06,2.26] | 1.39 | [1.33,1.45] | 1.33 | [1.27,1.39] |
| **Childhood social class** |  |  |  |  |  |  |  |  |  |  |
| Professional & managerial | 1 | [1.00,1.00] | 1 | [1.00,1.00] | 1 | [1.00,1.00] | 1 | [1.00,1.00] | 1 | [1.00,1.00] |
| Non-manual | 0.9 | [0.76,1.05] | 0.97 | [0.86,1.09] | 1.01 | [0.93,1.11] | 1.06 | [0.98,1.15] | 1.02 | [0.95,1.11] |
| Manual | 1.05 | [0.94,1.16] | 1.24 | [1.14,1.35] | 1.42 | [1.33,1.50] | 1.28 | [1.21,1.35] | 1.24 | [1.17,1.31] |
| Partly & unskilled | 1.09 | [0.97,1.23] | 1.34 | [1.23,1.47] | 1.47 | [1.38,1.58] | 1.31 | [1.23,1.39] | 1.25 | [1.18,1.33] |
| **Educational Level** |  |  |  |  |  |  |  |  |  |  |
| None |  |  |  |  |  |  | 1.95 | [1.79,2.13] | 2.23 | [2.04,2.44] |
| nvq1 level |  |  |  |  |  |  | 1.71 | [1.56,1.87] | 1.97 | [1.80,2.15] |
| nvq2 level |  |  |  |  |  |  | 1.58 | [1.47,1.70] | 1.74 | [1.61,1.87] |
| nvq3 level |  |  |  |  |  |  | 1.56 | [1.43,1.69] | 1.66 | [1.53,1.80] |
| nvq4 level |  |  |  |  |  |  | 1.47 | [1.36,1.59] | 1.55 | [1.43,1.67] |
| nvq5 level |  |  |  |  |  |  | 1 | [1.00,1.00] | 1 | [1.00,1.00] |
| **Adulthood social class** |  |  |  |  |  |  |  |  |  |  |
| Professional & managerial |  |  |  |  |  |  |  |  | 1 | [1.00,1.00] |
| Non-manual |  |  |  |  |  |  |  |  | 0.97 | [0.92,1.03] |
| Manual |  |  |  |  |  |  |  |  | 1.01 | [0.95,1.06] |
| Partly & unskilled |  |  |  |  |  |  |  |  | 0.95 | [0.90,1.01] |
| ***Healthy BMI & mental ill-health*** |  |  |  |  |  |  |  |  |  |  |
| Male | 1 | [1.00,1.00] | 1 | [1.00,1.00] | 1 | [1.00,1.00] | 1 | [1.00,1.00] | 1 | [1.00,1.00] |
| Female | 1.03 | [0.97,1.09] | 1.42 | [1.35,1.50] | 2.51 | [2.32,2.71] | 1.57 | [1.42,1.73] | 1.43 | [1.30,1.57] |
| **Cohort** |  |  |  |  |  |  |  |  |  |  |
| NCDS58 | 1 | [1.00,1.00] | 1 | [1.00,1.00] | 1 | [1.00,1.00] | 1 | [1.00,1.00] | 1 | [1.00,1.00] |
| BCS70 | 0.59 | [0.56,0.63] | 1.24 | [1.17,1.31] | 1.8 | [1.67,1.94] | 2.47 | [2.23,2.73] | 1.46 | [1.34,1.59] |
| **Childhood social class** |  |  |  |  |  |  |  |  |  |  |
| Professional & managerial | 1 | [1.00,1.00] | 1 | [1.00,1.00] | 1 | [1.00,1.00] | 1 | [1.00,1.00] | 1 | [1.00,1.00] |
| Non-manual | 1.13 | [1.01,1.26] | 1.21 | [1.09,1.34] | 1.35 | [1.16,1.56] | 0.87 | [0.72,1.05] | 0.97 | [0.82,1.14] |
| Manual | 1.14 | [1.05,1.23] | 1.22 | [1.14,1.31] | 1.56 | [1.41,1.73] | 1.19 | [1.04,1.35] | 1.06 | [0.95,1.19] |
| Partly & unskilled | 1.28 | [1.17,1.40] | 1.38 | [1.27,1.49] | 1.63 | [1.45,1.82] | 1.08 | [0.93,1.24] | 1.09 | [0.96,1.23] |
| **Educational Level** |  |  |  |  |  |  |  |  |  |  |
| None |  |  |  |  |  |  | 4.22 | [3.35,5.32] | 2.43 | [2.03,2.90] |
| nvq1 level |  |  |  |  |  |  | 2.57 | [2.01,3.27] | 1.85 | [1.54,2.22] |
| nvq2 level |  |  |  |  |  |  | 2.11 | [1.70,2.63] | 1.48 | [1.26,1.75] |
| nvq3 level |  |  |  |  |  |  | 1.59 | [1.24,2.04] | 1.21 | [1.01,1.45] |
| nvq4 level |  |  |  |  |  |  | 1.35 | [1.07,1.71] | 1.04 | [0.88,1.23] |
| nvq5 level |  |  |  |  |  |  | 1 | [1.00,1.00] | 1 | [1.00,1.00] |
| **Adulthood social class** |  |  |  |  |  |  |  |  |  |  |
| Professional & managerial |  |  |  |  |  |  |  |  | 1 | [1.00,1.00] |
| Non-manual |  |  |  |  |  |  |  |  | 1.26 | [1.14,1.39] |
| Manual |  |  |  |  |  |  |  |  | 1.23 | [1.10,1.37] |
| Partly & unskilled |  |  |  |  |  |  |  |  | 1.25 | [1.13,1.39] |
| ***Overweight/obese & mental ill-health*** |  |  |  |  |  |  |  |  |  |  |
| Male | 1 | [1.00,1.00] | 1 | [1.00,1.00] | 1 | [1.00,1.00] | 1 | [1.00,1.00] | 1 | [1.00,1.00] |
| Female | 1.37 | [1.16,1.60] | 1.68 | [1.49,1.88] | 1.52 | [1.36,1.69] | 1.04 | [0.95,1.13] | 0.86 | [0.79,0.93] |
| **Cohort** |  |  |  |  |  |  |  |  |  |  |
| NCDS58 | 1 | [1.00,1.00] | 1 | [1.00,1.00] | 1 | [1.00,1.00] | 1 | [1.00,1.00] | 1 | [1.00,1.00] |
| BCS70 | 0.59 | [0.50,0.70] | 1.85 | [1.64,2.08] | 3.4 | [3.00,3.84] | 3.95 | [3.56,4.38] | 2.35 | [2.18,2.53] |
| **Childhood social class** |  |  |  |  |  |  |  |  |  |  |
| Professional & managerial | 1 | [1.00,1.00] | 1 | [1.00,1.00] | 1 | [1.00,1.00] | 1 | [1.00,1.00] | 1 | [1.00,1.00] |
| Non-manual | 1.16 | [0.85,1.59] | 1.13 | [0.88,1.45] | 1.27 | [1.01,1.61] | 0.91 | [0.75,1.11] | 1.06 | [0.92,1.23] |
| Manual | 1.32 | [1.06,1.64] | 1.53 | [1.30,1.81] | 2.05 | [1.76,2.40] | 1.48 | [1.30,1.69] | 1.47 | [1.33,1.63] |
| Partly & unskilled | 1.4 | [1.10,1.78] | 2.14 | [1.79,2.55] | 2.4 | [2.04,2.83] | 1.56 | [1.36,1.80] | 1.69 | [1.51,1.89] |
| **Educational Level** |  |  |  |  |  |  |  |  |  |  |
| None |  |  |  |  |  |  | 5.9 | [4.51,7.73] | 4.45 | [3.73,5.31] |
| nvq1 level |  |  |  |  |  |  | 3.4 | [2.57,4.49] | 2.99 | [2.50,3.58] |
| nvq2 level |  |  |  |  |  |  | 2.74 | [2.11,3.55] | 2.34 | [1.99,2.76] |
| nvq3 level |  |  |  |  |  |  | 2.02 | [1.53,2.67] | 2.09 | [1.75,2.49] |
| nvq4 level |  |  |  |  |  |  | 1.9 | [1.46,2.48] | 1.69 | [1.43,2.00] |
| nvq5 level |  |  |  |  |  |  | 1 | [1.00,1.00] | 1 | [1.00,1.00] |
| **Adulthood social class** |  |  |  |  |  |  |  |  |  |  |
| Professional & managerial |  |  |  |  |  |  |  |  | 1 | [1.00,1.00] |
| Non-manual |  |  |  |  |  |  |  |  | 1.2 | [1.11,1.31] |
| Manual |  |  |  |  |  |  |  |  | 1.28 | [1.17,1.40] |
| Partly & unskilled |  |  |  |  |  |  |  |  | 1.21 | [1.11,1.32] |

**Supplemental Table 5. Relative risk ratios (RRR) between childhood social class and risk for i. overweight or obesity and good mental health, ii. Healthy BMI and mental ill-health and iii. Overweight or obesity and mental ill-health in 30 868 participants from the 1958 National Child Development Study and the 1970 British Cohort Study. Models stratified by cohort. Healthy BMI and normal mental health is the reference category**

|  | **Age 10** | | | | **Age 16** | | | | **Age 23** | | | |
| --- | --- | --- | --- | --- | --- | --- | --- | --- | --- | --- | --- | --- |
|  | **NCDS58** | | **BCS70** | | **NCDS58** | | **BCS70** | | **NCDS58** | | **BCS70** | |
|  | RRR | 95% CI | RRR | 95% CI | RRR | 95% CI | RRR | 95% CI | RRR | 95% CI | RRR | 95% CI |
| ***Healthy BMI & good MH*** | **Ref** |  | **Ref** |  | **Ref** |  | **Ref** |  | **Ref** |  | **Ref** |  |
| ***Overweight/Obese & good MH*** |  |  |  |  |  |  |  |  |  |  |  |  |
| **Childhood social class** |  |  |  |  |  |  |  |  |  |  |  |  |
| Professional/managerial | 1 |  | 1 |  | 1 |  | 1 |  | 1 |  | 1 |  |
| Non-manual | 0.76 | [0.58,1.00] | 0.98 | [0.74,1.30] | 0.94 | [0.73,1.20] | 1.08 | [0.82,1.42] | 1.11 | [0.91,1.34] | 0.98 | [0.83,1.17] |
| Manual | 0.98 | [0.83,1.17] | 1.09 | [0.91,1.31] | **1.27** | **[1.09,1.50]** | **1.27** | **[1.07,1.50]** | **1.76** | **[1.54,2.01]** | **1.3** | **[1.15,1.47]** |
| Partly & unskilled | 0.96 | [0.79,1.17] | **1.29** | **[1.06,1.57]** | **1.27** | **[1.06,1.53]** | **1.51** | **[1.24,1.84]** | **1.8** | **[1.56,2.07]** | **1.36** | **[1.17,1.59]** |
| **Educational level** |  |  |  |  |  |  |  |  |  |  |  |  |
| None |  |  |  |  |  |  |  |  |  |  |  |  |
| Nvq 1 level |  |  |  |  |  |  |  |  |  |  |  |  |
| Nvq 2 level |  |  |  |  |  |  |  |  |  |  |  |  |
| Nvq 3 level |  |  |  |  |  |  |  |  |  |  |  |  |
| Nvq 4 level |  |  |  |  |  |  |  |  |  |  |  |  |
| Nvq 5 level |  |  |  |  |  |  |  |  |  |  |  |  |
| **Adulthood social class** |  |  |  |  |  |  |  |  |  |  |  |  |
| Professional/managerial |  |  |  |  |  |  |  |  |  |  |  |  |
| Non-manual |  |  |  |  |  |  |  |  |  |  |  |  |
| Manual |  |  |  |  |  |  |  |  |  |  |  |  |
| Partly & unskilled |  |  |  |  |  |  |  |  |  |  |  |  |
| ***Healthy BMI & mental ill-health*** |  |  |  |  |  |  |  |  |  |  |  |  |
| **Childhood social class** |  |  |  |  |  |  |  |  |  |  |  |  |
| Professional/managerial | 1 |  | 1 |  | 1 |  | 1 |  | 1 |  | 1 |  |
| Non-manual | 1.14 | [0.98,1.32] | 1.04 | [0.86,1.25] | 1.16 | [0.96,1.39] | **1.25** | **[1.02,1.53]** | 1.21 | [0.93,1.58] | **1.38** | **[1.09,1.74]** |
| Manual | 1.07 | [0.96,1.19] | **1.24** | **[1.10,1.41]** | **1.16** | **[1.02,1.33]** | **1.31** | **[1.14,1.51]** | **1.62** | **[1.34,1.95]** | **1.49** | **[1.25,1.79]** |
| Partly & unskilled | **1.15** | **[1.02,1.29]** | **1.43** | **[1.24,1.66]** | **1.24** | **[1.07,1.44]** | **1.49** | **[1.27,1.76]** | **1.88** | **[1.54,2.29]** | **1.45** | **[1.18,1.79]** |
| **Educational level** |  |  |  |  |  |  |  |  |  |  |  |  |
| None |  |  |  |  |  |  |  |  |  |  |  |  |
| Nvq 1 level |  |  |  |  |  |  |  |  |  |  |  |  |
| Nvq 2 level |  |  |  |  |  |  |  |  |  |  |  |  |
| Nvq 3 level |  |  |  |  |  |  |  |  |  |  |  |  |
| Nvq 4 level |  |  |  |  |  |  |  |  |  |  |  |  |
| Nvq 5 level |  |  |  |  |  |  |  |  |  |  |  |  |
| **Adulthood social class** |  |  |  |  |  |  |  |  |  |  |  |  |
| Professional/managerial |  |  |  |  |  |  |  |  |  |  |  |  |
| Non-manual |  |  |  |  |  |  |  |  |  |  |  |  |
| Manual |  |  |  |  |  |  |  |  |  |  |  |  |
| Partly & unskilled |  |  |  |  |  |  |  |  |  |  |  |  |
| ***Overweight/obese & mental ill-health*** |  |  |  |  |  |  |  |  |  |  |  |  |
| **Childhood social class** |  |  |  |  |  |  |  |  |  |  |  |  |
| Professional/managerial | 1 |  | 1 |  | 1 |  | 1 |  | 1 |  | 1 |  |
| Non-manual | 0.95 | [0.61,1.49] | 1.22 | [0.66,2.28] | 0.86 | [0.47,1.59] | 1.21 | [0.74,1.95] | 1.32 | [0.70,2.48] | 1.15 | [0.79,1.69] |
| Manual | 1.18 | [0.87,1.60] | 1.47 | [0.95,2.27] | 1.36 | [0.91,2.04] | **1.56** | **[1.12,2.18]** | **2.76** | **[1.79,4.24]** | **1.84** | **[1.42,2.37]** |
| Partly & unskilled | 1.16 | [0.82,1.62] | **2.01** | **[1.29,3.14]** | **1.71** | **[1.12,2.61]** | **2.33** | **[1.63,3.32]** | **3.05** | **[1.91,4.88]** | **2.12** | **[1.56,2.89]** |
| **Educational level** |  |  |  |  |  |  |  |  |  |  |  |  |
| None |  |  |  |  |  |  |  |  |  |  |  |  |
| Nvq 1 level |  |  |  |  |  |  |  |  |  |  |  |  |
| Nvq 2 level |  |  |  |  |  |  |  |  |  |  |  |  |
| Nvq 3 level |  |  |  |  |  |  |  |  |  |  |  |  |
| Nvq 4 level |  |  |  |  |  |  |  |  |  |  |  |  |
| Nvq 5 level |  |  |  |  |  |  |  |  |  |  |  |  |
| **Adulthood social class** |  |  |  |  |  |  |  |  |  |  |  |  |
| Professional/managerial |  |  |  |  |  |  |  |  |  |  |  |  |
| Non-manual |  |  |  |  |  |  |  |  |  |  |  |  |
| Manual |  |  |  |  |  |  |  |  |  |  |  |  |
| Partly & unskilled |  |  |  |  |  |  |  |  |  |  |  |  |

**Supplemental Table 5, continued**

|  | | | **Age 34** | | | | **Age 42** | | | | | | |
| --- | --- | --- | --- | --- | --- | --- | --- | --- | --- | --- | --- | --- | --- |
|  | | | **NCDS58** | | **BCS70** | | **NCDS58** | | **BCS70** | | | | |
|  | | | RRR | 95% CI | RRR | 95% CI | RRR | 95% CI | RRR | | | | 95% CI |
| ***Healthy BMI & good MH*** | | | **Ref** |  | **Ref** |  | **Ref** |  | **Ref** | | | |  |
| ***Overweight/Obese & good MH*** | | |  |  |  |  |  |  |  | | | |  |
| **Childhood social class** | | |  |  |  |  |  |  |  | | | |  |
| Professional/managerial | | | 1 |  | 1 |  | 1 |  | 1 | | | |  |
| Non-manual | | | **1.16** | **[1.01,1.34]** | 0.93 | [0.81,1.07] | **1.15** | **[1.00,1.33]** | 0.89 | | | | [0.76,1.03] |
| Manual | | | **1.28** | **[1.16,1.41]** | **1.30** | **[1.17,1.46]** | **1.23** | **[1.11,1.36]** | **1.27** | | | | **[1.14,1.43]** |
| Partly & unskilled | | | **1.27** | **[1.14,1.42]** | **1.38** | **[1.20,1.60]** | **1.24** | **[1.10,1.41]** | **1.25** | | | | **[1.09,1.44]** |
| **Educational level** | | |  |  |  |  |  |  |  | | | |  |
| None | | | **1.94** | **[1.63,2.31]** | **2.18** | **[1.75,2.71]** | **2.35** | **[1.94,2.86]** | **2.23** | | | | **[1.74,2.86]** |
| Nvq 1 level | | | **1.78** | **[1.49,2.12]** | **1.88** | **[1.50,2.36]** | **2.10** | **[1.73,2.54]** | **2.09** | | | | **[1.57,2.77]** |
| Nvq 2 level | | | **1.56** | **[1.34,1.81]** | **1.87** | **[1.53,2.30]** | **1.75** | **[1.49,2.07]** | **1.93** | | | | **[1.53,2.45]** |
| Nvq 3 level | | | **1.50** | **[1.27,1.77]** | **1.80** | **[1.45,2.24]** | **1.54** | **[1.30,1.84]** | **1.99** | | | | **[1.51,2.63]** |
| Nvq 4 level | | | **1.51** | **[1.27,1.80]** | **1.64** | **[1.34,2.00]** | **1.66** | **[1.39,1.98]** | **1.64** | | | | **[1.30,2.06]** |
| Nvq 5 level | | | 1 |  | 1 |  | 1 |  | 1 | | | |  |
| **Childhood social class** | | |  |  |  |  |  |  |  | | | |  |
| Professional/managerial | | |  |  |  |  | 1 |  | 1 | | | |  |
| Non-manual | | |  |  |  |  | 0.85 | [0.75,0.96] | 1.13 | | | | [0.99,1.29] |
| Manual | | |  |  |  |  | 0.91 | [0.79,1.04] | 1.14 | | | | [0.96,1.36] |
| Partly & unskilled | | |  |  |  |  | 0.91 | [0.79,1.05] | 1.05 | | | | [0.88,1.27] |
| ***Healthy BMI & mental ill-health*** | | |  |  |  |  |  |  |  | | | |  |
| **Childhood social class** | | |  |  |  |  |  |  |  | | | |  |
| Professional/managerial | | | 1 |  | 1 |  | 1 |  | 1 | | | |  |
| Non-manual | | | 0.91 | [0.62,1.35] | 0.82 | [0.61,1.12] | 0.85 | [0.63,1.16] | 1.03 | | | | [0.79,1.35] |
| Manual | | | 1.09 | [0.85,1.41] | **1.21** | **[1.00,1.47]** | 1.03 | [0.82,1.28] | 1.19 | | | | [0.95,1.48] |
| Partly & unskilled | | | 1.10 | [0.83,1.46] | 1.12 | [0.88,1.44] | 1.01 | [0.79,1.29] | 1.19 | | | | [0.91,1.56] |
| **Educational level** | | |  |  |  |  |  |  |  | | | |  |
| None | | | **6.32** | **[4.10,9.75]** | **2.46** | **[1.62,3.72]** | **2.71** | **[1.87,3.93]** | **1.99** | | | | **[1.21,3.29]** |
| Nvq 1 level | | | **3.12** | **[1.96,4.97]** | **1.96** | **[1.27,3.04]** | **1.67** | **[1.12,2.49]** | **2.16** | | | | **[1.31,3.55]** |
| Nvq 2 level | | | **2.23** | **[1.46,3.41]** | **1.89** | **[1.28,2.79]** | 1.25 | [0.90,1.76] | **1.66** | | | | **[1.07,2.57]** |
| Nvq 3 level | | | 1.62 | [0.98,2.69] | 1.40 | [0.93,2.09] | 1.14 | [0.75,1.71] | 1.31 | | | | [0.83,2.09] |
| Nvq 4 level | | | 1.51 | [0.92,2.45] | 1.18 | [0.80,1.73] | 1.02 | [0.71,1.45] | 1.09 | | | | [0.70,1.69] |
| Nvq 5 level | | | 1 |  | 1 |  | 1 |  | 1 | | | |  |
| **Adulthood social class** | | |  |  |  |  |  |  |  | | | |  |
| Professional/managerial | | |  |  |  |  | 1 |  | 1 | | | |  |
| Non-manual | | |  |  |  |  | 1.28 | [0.99,1.66] | 1.19 | | | | [0.94,1.51] |
| Manual | | |  |  |  |  | 1.33 | [0.99,1.77] | 1.19 | | | | [0.88,1.63] |
| Partly & unskilled | | |  |  |  |  | 1.17 | [0.89,1.54] | 1.26 | | | | [0.97,1.64] |
| ***Overweight/obese & mental ill-health*** | | |  |  |  |  |  |  |  | | | |  |
| **Childhood social class** | | |  |  |  |  |  |  |  | | | |  |
| Professional/managerial | | | 1 |  | 1 |  | 1 |  | 1 | | | |  |
| Non-manual | | | 1.36 | [0.84,2.18] | 0.82 | [0.60,1.11] | 1 | [0.73,1.36] | 1.14 | | | | [0.87,1.48] |
| Manual | | | **1.64** | **[1.18,2.27]** | **1.50** | **[1.23,1.82]** | **1.35** | **[1.10,1.67]** | **1.60** | | | | **[1.34,1.91]** |
| Partly & unskilled | | | **1.57** | **[1.09,2.25]** | **1.69** | **[1.36,2.10]** | **1.52** | **[1.21,1.90]** | **1.81** | | | | **[1.47,2.23]** |
| **Educational level** | | |  |  |  |  |  |  |  | | | |  |
| None | | | **9.31** | **[5.39,16.09]** | **4.41** | **[2.82,6.91]** | **5.93** | **[3.89,9.03]** | **3.58** | | | | **[2.33,5.49]** |
| Nvq 1 level | | | **3.92** | **[2.21,6.96]** | **3.34** | **[2.14,5.22]** | **3.01** | **[1.96,4.63]** | **3.46** | | | | **[2.24,5.35]** |
| Nvq 2 level | | | **2.48** | **[1.46,4.20]** | **2.91** | **[1.91,4.42]** | **2.13** | **[1.43,3.18]** | **2.57** | | | | **[1.68,3.93]** |
| Nvq 3 level | | | 1.52 | [0.82,2.83] | **2.13** | **[1.37,3.33]** | **1.83** | **[1.22,2.74]** | **2.41** | | | | **[1.58,3.69]** |
| Nvq 4 level | | | 1.71 | [0.94,3.14] | **1.87** | **[1.25,2.80]** | **1.66** | **[1.13,2.43]** | **1.75** | | | | **[1.20,2.56]** |
| Nvq 5 level | | | 1 |  | 1 |  | 1 |  | 1 | | | |  |
| **Adulthood social class** | | |  |  |  |  |  |  |  | | | |  |
| Professional/managerial | | |  |  |  |  | 1 |  | 1 | | | |  |
| Non-manual | | |  |  |  |  | 1.06 | [0.82,1.37] | **1.30** | | | | **[1.05,1.62]** |
| Manual | | |  |  |  |  | 1.08 | [0.84,1.40] | **1.39** | | | | **[1.11,1.74]** |
| Partly & unskilled | | |  |  |  |  | 0.99 | [0.75, 1.30] | 1.38 | | | | 1.10, 1.73] |
|  | |  |  |  |  |  |  | | | |  |  |  |

**Supplemental Table 6. Relative risk ratios (RRR) between childhood social class and risk for i. overweight or obesity and good mental health, ii. Healthy BMI and mental ill-health and iii. Overweight or obesity and mental ill-health in 30 868 participants from the 1958 National Child Development Study and the 1970 British Cohort Study. Healthy BMI and normal mental health is the reference category**

|  | **Age 10/11** | | | | **Age 16** | | | | **Age 23** | | | |
| --- | --- | --- | --- | --- | --- | --- | --- | --- | --- | --- | --- | --- |
|  | **RRR** | **95% CI** | **RRR** | **95% CI** | **RRR** | **95% CI** | **RRR** | **95% CI** | **RRR** | **95% CI** | **RRR** | **95% CI** |
|  |  | **NCDS 58** |  | **BCS 70** |  | **NCDS 58** |  | **BCS 70** |  | **NCDS 58** |  | **BCS 70** |
| ***Healthy BMI & good MH*** | **Ref** |  | **Ref** |  | **Ref** |  | **Ref** |  | **Ref** |  | **Ref** |  |
| ***Overweight/Obese & good MH*** |  |  |  |  |  |  |  |  |  |  |  |  |
| Male | 1 |  | 1 |  | 1 |  | 1 |  | 1 |  | 1 |  |
| Female | **1.38** | **[1.21,1.57]** | **1.64** | **[1.40,1.91]** | **1.49** | **[1.32,1.69]** | **1.15** | **[1.02,1.30]** | **0.66** | **[0.60,0.72]** | **0.54** | **[0.49,0.59]** |
| **Childhood social class** |  |  |  |  |  |  |  |  |  |  |  |  |
| Professional & managerial | 1 |  | 1 |  | 1 |  | 1 |  | 1 |  | 1 |  |
| Non-manual | 0.76 | [0.58,1.00] | 0.98 | [0.74,1.30] | 0.94 | [0.73,1.20] | 1.08 | [0.82,1.42] | **1.11** | **[0.91,1.34]** | 0.98 | [0.83,1.17] |
| Manual | 0.98 | [0.83,1.17] | 1.09 | [0.91,1.31] | **1.27** | **[1.09,1.50]** | **1.27** | **[1.07,1.50]** | **1.76** | **[1.54,2.01]** | **1.3** | **[1.15,1.47]** |
| Partly & unskilled | 0.96 | [0.79,1.17] | **1.29** | **[1.06,1.57]** | **1.27** | **[1.06,1.53]** | **1.51** | **[1.24,1.84]** | **1.8** | **[1.56,2.07]** | **1.36** | **[1.17,1.59]** |
| ***Normal BMI & mental ill-health*** |  |  |  |  |  |  |  |  |  |  |  |  |
| Male | 1 |  | 1 |  | 1 |  | 1 |  | 1 |  | 1 |  |
| Female | 1.04 | [0.96,1.13] | 1.01 | [0.91,1.12] | **1.4** | **[1.27,1.53]** | **1.4** | **[1.26,1.55]** | **3.1** | **[2.68,3.60]** | **2.01** | **[1.72,2.35]** |
| **Childhood social class** |  |  |  |  |  |  |  |  |  |  |  |  |
| Professional & managerial | 1 |  | 1 |  | 1 |  | 1 |  | 1 |  | 1 |  |
| Non-manual | 1.14 | [0.98,1.32] | 1.04 | [0.86,1.25] | 1.16 | [0.96,1.39] | **1.25** | **[1.02,1.53]** | 1.21 | [0.93,1.58] | **1.38** | **[1.09,1.74]** |
| Manual | 1.07 | [0.96,1.19] | **1.24** | **[1.10,1.41]** | **1.16** | **[1.02,1.33]** | **1.31** | **[1.14,1.51]** | **1.62** | **[1.34,1.95]** | **1.49** | **[1.25,1.79]** |
| Partly & unskilled | **1.15** | **[1.02,1.29]** | **1.43** | **[1.24,1.66]** | **1.24** | **[1.07,1.44]** | **1.49** | **[1.27,1.76]** | **1.88** | **[1.54,2.29]** | **1.45** | **[1.18,1.79]** |
| ***Overweight/obese & mental ill-health*** |  |  |  |  |  |  |  |  |  |  |  |  |
| Male | 1 |  | 1 |  | 1 |  | 1 |  | 1 |  | 1 |  |
| Female | **1.29** | **[1.02,1.63]** | 1.33 | [0.98,1.81] | **1.88** | **[1.42,2.47]** | **1.45** | **[1.12,1.87]** | **2.14** | **[1.64,2.81]** | 1.21 | [0.99,1.47] |
| **Childhood social class** |  |  |  |  |  |  |  |  |  |  |  |  |
| Professional & managerial | 1 |  | 1 |  | 1 |  | 1 |  | 1 |  | 1 |  |
| Non-manual | 0.95 | [0.61,1.49] | 1.22 | [0.66,2.28] | 0.86 | [0.47,1.59] | 1.21 | [0.74,1.95] | 1.32 | [0.70,2.48] | 1.15 | [0.79,1.69] |
| Manual | 1.18 | [0.87,1.60] | 1.47 | [0.95,2.27] | 1.36 | [0.91,2.04] | **1.56** | **[1.12,2.18]** | **2.76** | **[1.79,4524]** | **1.84** | **[1.42,2.37]** |
| Partly & unskilled | 1.16 | [0.82,1.62] | **2.01** | **[1.29,3.14]** | **1.71** | **[1.12,2.61]** | **2.33** | **[1.63,3.32]** | **3.05** | **[1.91,4.88]** | **2.12** | **[1.56,2.89]** |

**Supplemental Table 6. Continued.**

|  | **Age 34/33** | | | | **Age 42** | | | |
| --- | --- | --- | --- | --- | --- | --- | --- | --- |
|  | **RRR** | **95% CI** | **RRR** | **95% CI** | **RRR** | **95% CI** | **RRR** | **95% CI** |
|  |  | **NCDS 58** |  | **BCS 70** |  | **NCDS 58** |  | **BCS 70** |
| ***Healthy BMI & good MH*** | **Ref** |  | **Ref** |  | **Ref** |  | **Ref** |  |
| ***Overweight/Obese & good mental health*** |  |  |  |  |  |  |  |  |
| Male | 1 |  | 1 |  | 1 |  | 1 |  |
| Female | **0.54** | **[0.49,0.59]** | **0.53** | **[0.49,0.57]** | **0.48** | **[0.44,0.52]** | **0.5** | **[0.46,0.54]** |
| **Childhood social class** |  |  |  |  |  |  |  |  |
| Professional & managerial | 1 |  | 1 |  | 1 |  | 1 |  |
| Non-manual | 0.98 | [0.83,1.17] | **1.22** | **[1.06,1.40]** | 0.96 | [0.84,1.10] | **1.2** | **[1.04,1.39]** |
| Manual | **1.3** | **[1.15,1.47]** | **1.43** | **[1.30,1.58]** | **1.4** | **[1.26,1.56]** | **1.39** | **[1.27,1.54]** |
| Partly & unskilled | **1.36** | **[1.17,1.59]** | **1.47** | **[1.32,1.63]** | **1.51** | **[1.31,1.74]** | **1.47** | **[1.31,1.64]** |
| ***Normal BMI & mental ill-health*** |  |  |  |  |  |  |  |  |
| Male | 1 |  | 1 |  | 1 |  | 1 |  |
| Female | **2.01** | **[1.72,2.35]** | **2.15** | **[1.76,2.63]** | **1.4** | **[1.19,1.65]** | **1.84** | **[1.56,2.17]** |
| **Childhood social class** |  |  |  |  |  |  |  |  |
| Professional & managerial | 1 |  | 1 |  | 1 |  | 1 |  |
| Non-manual | **1.38** | **[1.09,1.74]** | 1.03 | [0.70,1.52] | 0.87 | [0.64,1.17] | 0.93 | [0.69,1.26] |
| Manual | **1.49** | **[1.25,1.79]** | **1.54** | **[1.21,1.97]** | **1.39** | **[1.16,1.68]** | **1.29** | **[1.05,1.58]** |
| Partly & unskilled | **1.45** | **[1.18,1.79]** | **1.76** | **[1.34,2.30]** | **1.33** | **[1.05,1.69]** | **1.38** | **[1.10,1.72]** |
| ***Overweight/obese & mental ill-health*** |  |  |  |  |  |  |  |  |
| Male | 1 |  | 1 |  | 1 |  | 1 |  |
| Female | 1.21 | [0.99,1.47] | **1.36** | **[1.10,1.69]** | 0.91 | [0.78,1.05] | 0.94 | [0.81,1.09] |
| **Childhood social class** |  |  |  |  |  |  |  |  |
| Professional & managerial | 1 |  | 1 |  | 1 |  | 1 |  |
| Non-manual | 1.15 | [0.79,1.69] | 1.58 | [0.98,2.53] | 0.87 | [0.65,1.18] | 1.12 | [0.82,1.53] |
| Manual | **1.84** | **[1.42,2.37]** | **2.51** | **[1.83,3.45]** | **1.78** | **[1.47,2.16]** | **1.86** | **[1.52,2.27]** |
| Partly & unskilled | **2.12** | **[1.56,2.89]** | **2.8** | **[1.99,3.96]** | **2.1** | **[1.70,2.60]** | **2.33** | **[1.87,2.89]** |

**Supplemental Figure 2 A, B, C & D. Relative risk ratios (RRR) for i. overweight or obesity and good mental health, ii. Healthy BMI and mental ill-health and iii. Overweight or obesity and mental ill-health in 30 868 participants from the 1958 National Child Development Study and the 1970 British Cohort Study, stratified by cohort. Healthy BMI and good mental health is the reference category**

**A. Age 11/10**

**
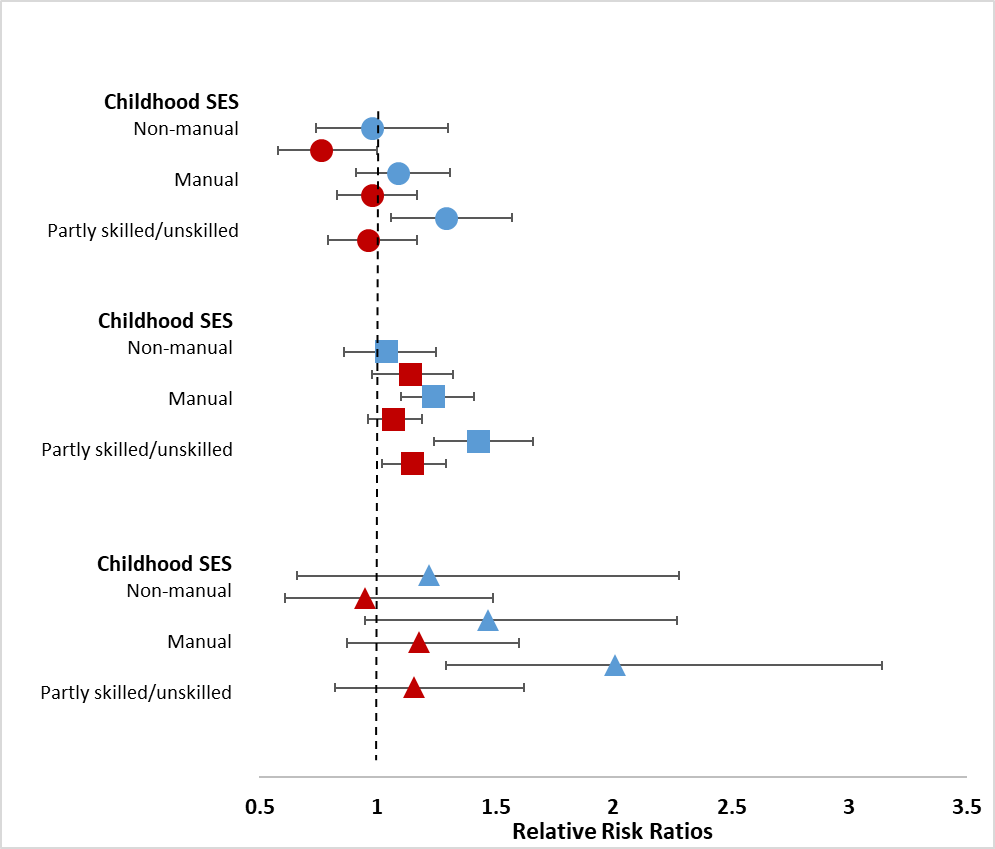
**

Reference category for childhood social class is the professional/managerial group.

**Figure legend:**

**
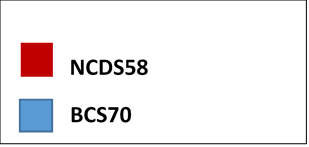
**

**B. Age 23**

**
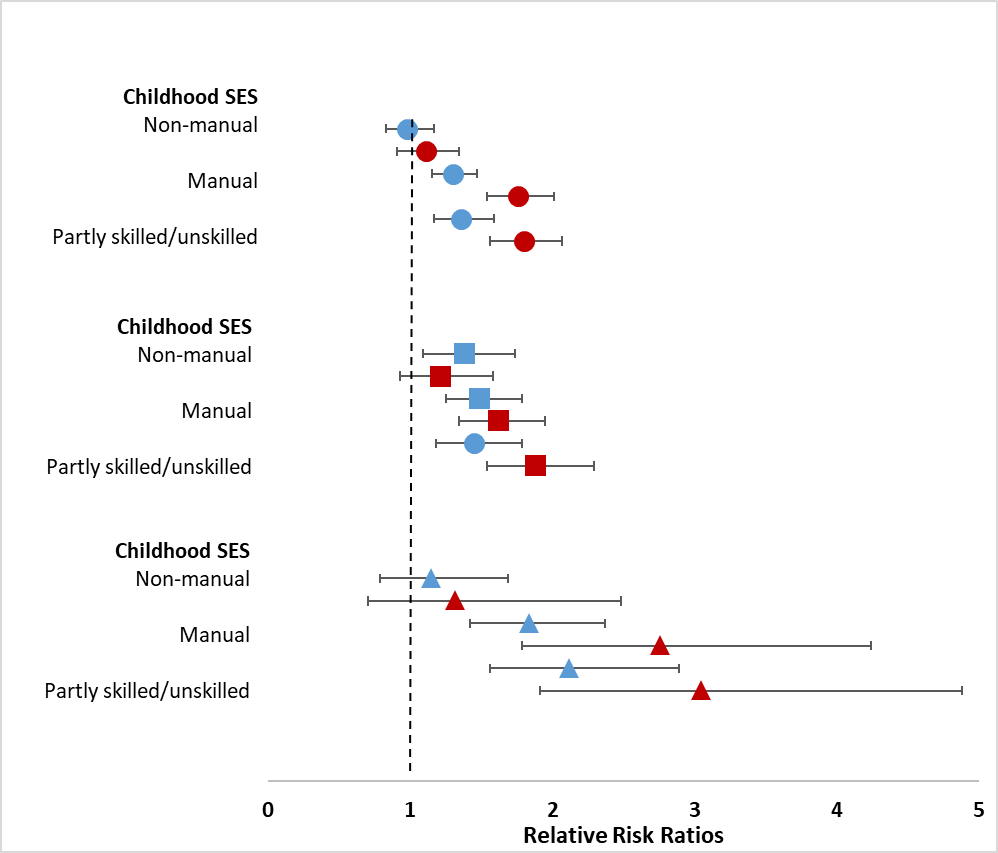
**

**NCDS58**

**BCS70**

Reference category for childhood social class is the professional/managerial group. **C. Age 34/33**

**
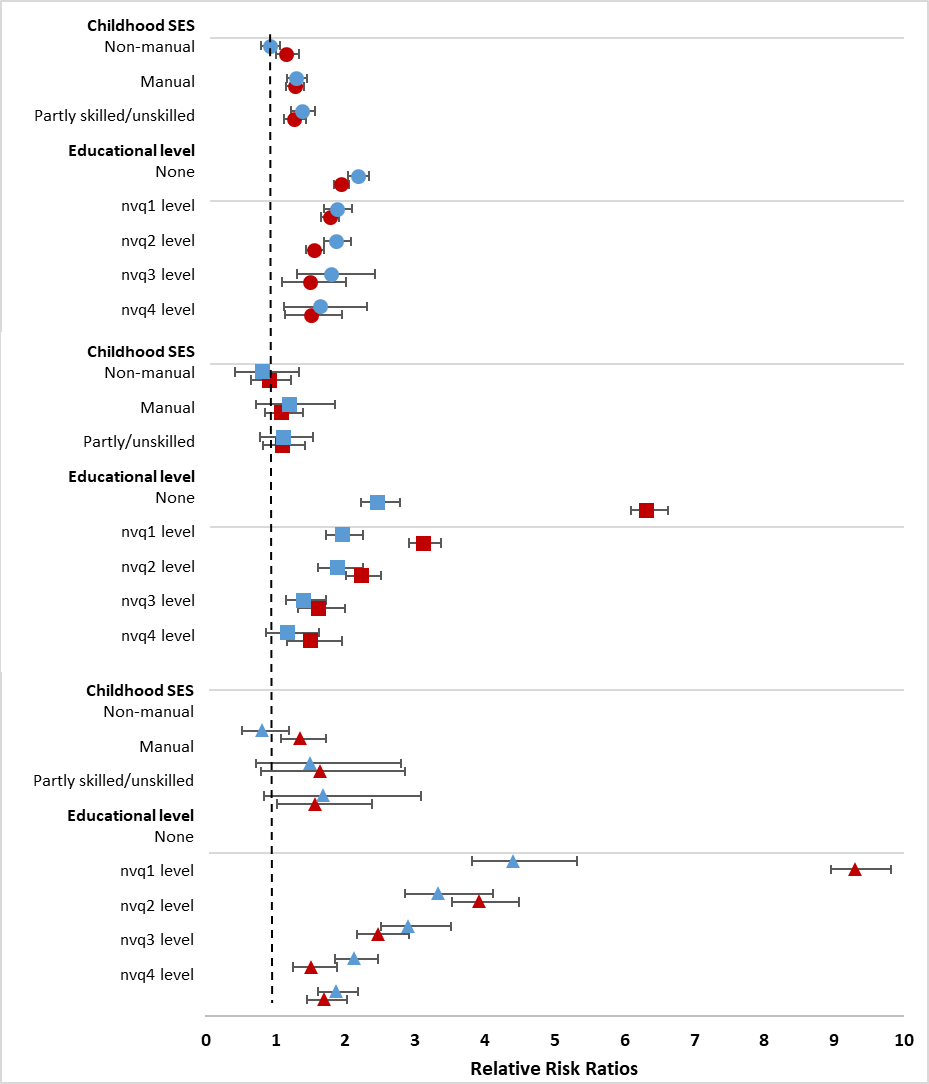

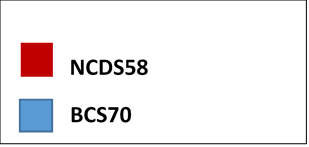
**

Reference category for childhood social class is the professional/managerial group. Reference category for educational level is NVQ 5 level (university degree).

**D. Age 42**

**
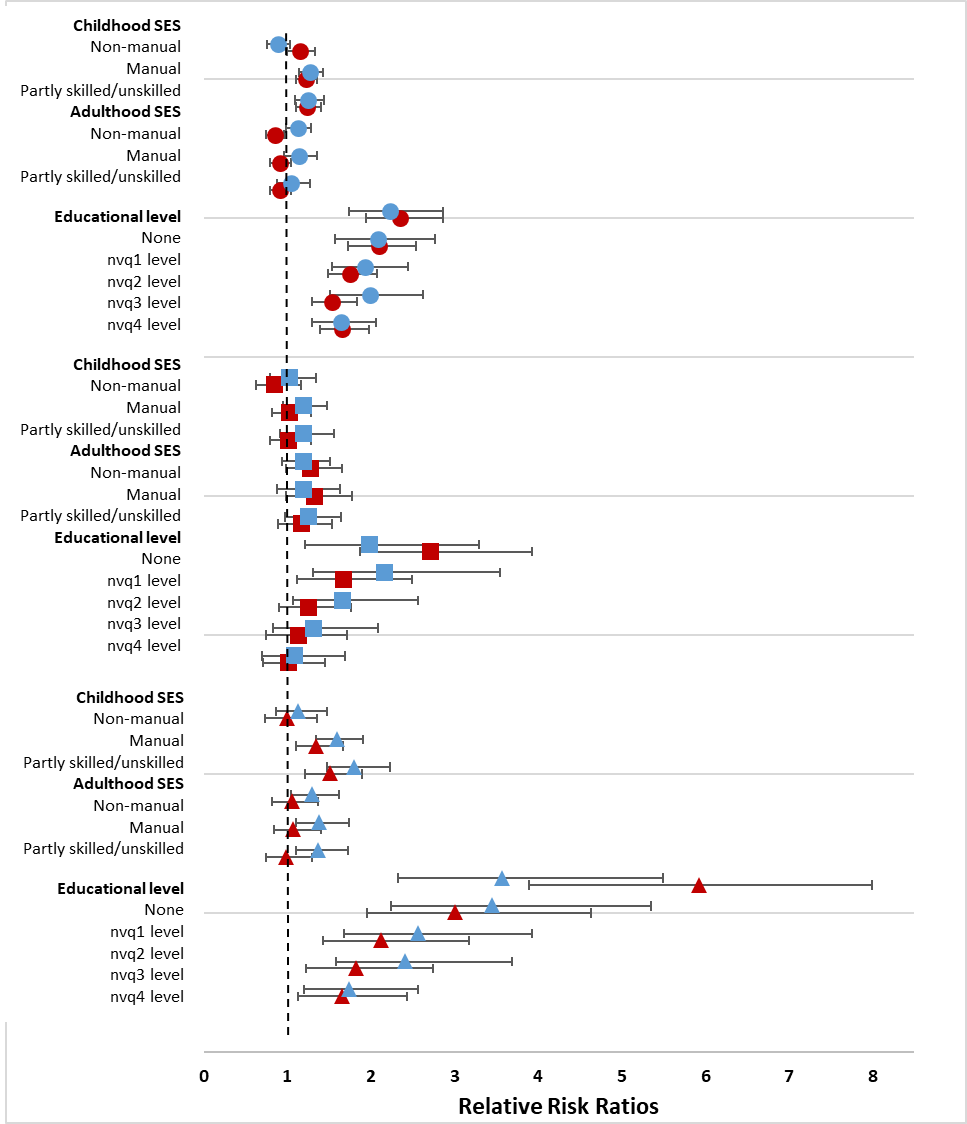
**

Reference category for childhood and adulthood social class is the professional/managerial group. Reference category for educational level is NVQ 5 level (university degree).

**Figure legend:**


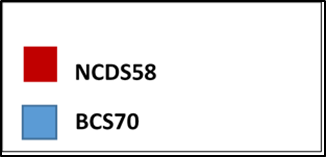

Supplement: Supplementary file 1 [file mmc1.docx]
